# Chemical constituents from the medicinal herb-derived fungus *Chaetomium globosum* Km1226

**DOI:** 10.1186/s40529-023-00406-8

**Published:** 2023-11-30

**Authors:** Chia-Hao Chang, George Hsiao, Shih-Wei Wang, Juei-Yu Yen, Shu-Jung Huang, Wei-Chiung Chi, Tzong-Huei Lee

**Affiliations:** 1 Institute of Fisheries Science, College of Life Science, National Taiwan University, Taipei, 10617 Taiwan; 2 Department of Pharmacology, School of Medicine, College of Medicine, Taipei Medical University, Taipei, 11031 Taiwan; 3 Graduate Institute of Medical Sciences, College of Medicine, Taipei Medical University, Taipei, 11031 Taiwan; 4 Department of Medicine, MacKay Medical College, New Taipei City, 25245 Taiwan; 5 Institute of Biomedical Sciences, Mackay Medical College, New Taipei City, 25245 Taiwan; 6 School of Pharmacy, College of Pharmacy, Kaohsiung, 807378 Taiwan; 7 Department of Chinese Medicine, MacKay Memorial Hospital, Taipei, 10449 Taiwan; 8 Department of Chinese Medicine, MacKay Memorial Hospital, Taipei, 10491 Taiwan; 9 Department of Food Science, National Quemoy University, Kinmen, 89250 Taiwan

**Keywords:** *Chaetomium globosum*, Aureonitol, Mollipilin, Polyketide, Anti-inflammation, Anti-angiogenesis

## Abstract

**Background:**

Endophytic fungi have proven to be a rich source of novel natural products with a wide-array of biological activities and higher levels of structural diversity.

**Results:**

Chemical investigation on the liquid- and solid-state fermented products of *Chaetomium globosum* Km1226 isolated from the littoral medicinal herb *Atriplex maximowicziana* Makino resulted in the isolation of compounds **1**–**14**. Their structures were determined by spectroscopic analysis as three previously undescribed C_13_-polyketides, namely aureonitol C (**1**), mollipilins G (**2**), and H (**3**), along with eleven known compounds **4**–**14**. Among these, mollipilin A (**5**) exhibited significant nitric oxide production inhibitory activity in LPS-induced BV-2 microglial cells with an IC_50_ value of 0.7 ± 0.1 µM, and chaetoglobosin D (**10**) displayed potent anti-angiogenesis property in human endothelial progenitor cells (EPCs) with an IC_50_ value of 0.8 ± 0.3 µM.

**Conclusions:**

Three previously unreported compounds **1**–**3** were isolated and identified. Mollipilin A (**5**) and chaetoglobosin D (**10**) could possibly be developed as anti-inflammatory and anti-angiogenic lead drugs, respectively.

**Supplementary Information:**

The online version contains supplementary material available at 10.1186/s40529-023-00406-8.

## Background

Endophytes are microorganisms that spend part or whole of their life cycles inside the healthy tissues of host plants, typically causing no apparent symptoms of diseases (Debbab et al. 2011; Kaul et al. 2012; Wang et al. 2017a; Ancheeva et al. 2020). In the symbiotic relationship, the endophytes are fed and protected by the host plants, and in return, the microorganisms produce bioactive secondary metabolites involving in the growth of the host plant and protecting the plants from pathogens and herbivores (Abdel-Azeem et al. 2016; Yang et al. 2021; Zhang et al. 2021; Collinge et al. 2022). The fungi, one major category of the endophytes, have been reported to be capable of producing a diverse array of specialized metabolites, and can produce chemical entities that are also present in plants or have similar biological activities as the compounds from plant sources (Mishra et al. 2022; Wen et al. 2022). Therefore, natural products derived from endophytic fungi were considered to be indispensable sources of new drugs with particular significance in lead structure discovery, due to their wide structural diversity and biological activities (Kumar et al. 2019; Qi et al. 2020; Song et al. 2020; Hridoy et al. 2022; Tiwari et al. 2022).

**Fig. 1:**
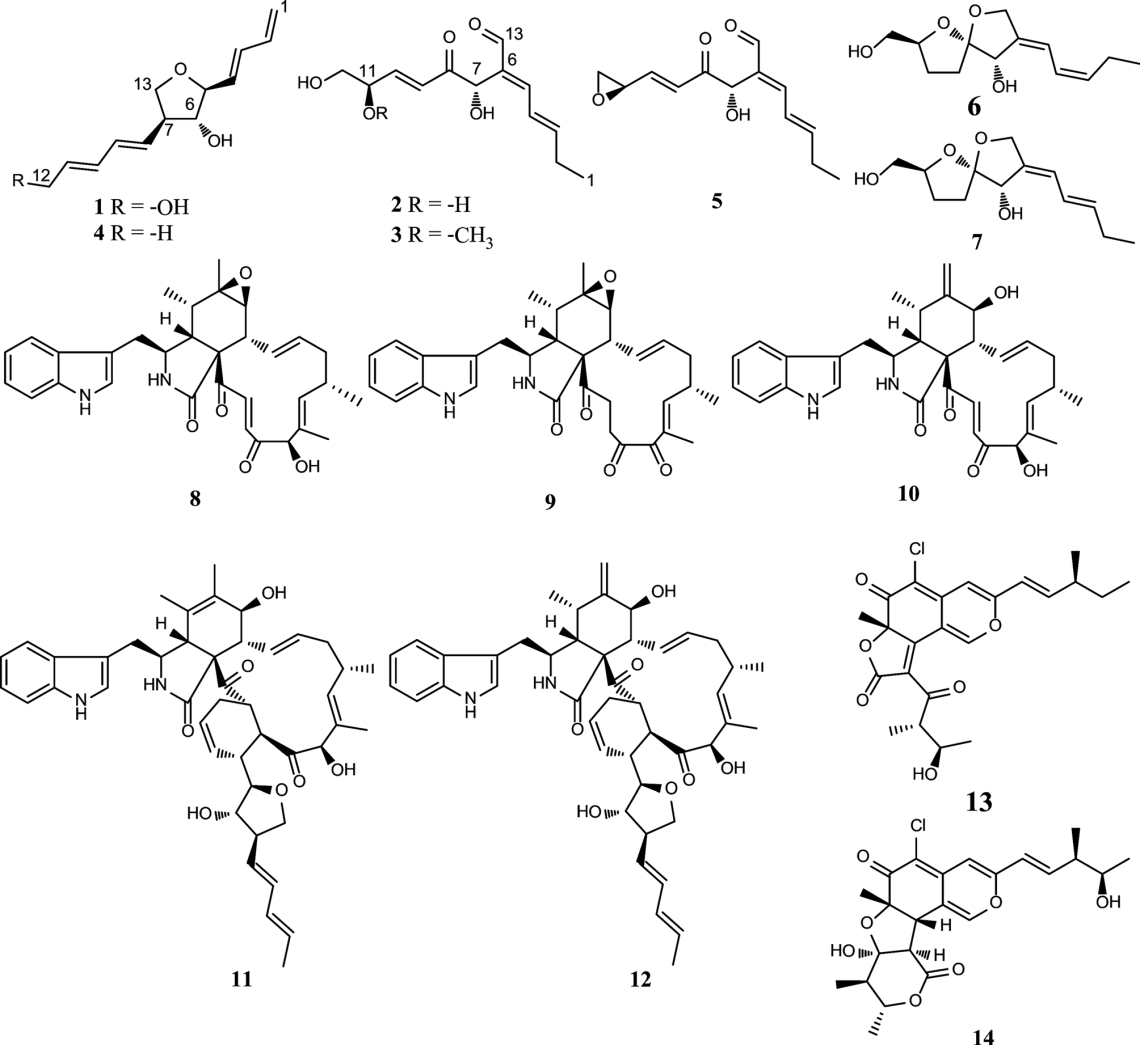
Chemical structures of compounds **1**–**14** identified in this study

The medicinal herb *Atriplex maximowicziana* Makino (Chenopodiaceae), also known as Hae-Fwu-Rong, distributed widely at sandy and coral-rocky seashores of East Asia, and the whole plant has long been used in folk remedies for dispelling pathogenic wind and treating rheumatoid arthritis (Chang et al. 2022). Since the production scale of this herb was not sufficient for commercialization, its associated microorganisms could possibly be an alternative source of the effective specialized compounds. In this study, the fungal strain *Chaetomium globosum* Km1226 (Chaetomiaceae) was isolated from the leaves of *A. maximowicziana* Makino. *C. globosum* Km1226 cultured in PDY media furnished the crude extracts that exhibited significant antimicrobial activity at a concentration of 1.0 mg/mL in a preliminary biological evaluation. These results prompted the isolation and identification of fourteen constituents including seven C_13_-polyketides including aureonitol C (**1**), mollipilins H and G (**2** and **3**), (–)-aureonitol (**4**), mollipilins A, E, and F (**5**–**7**), together with five cytochalasan alkaloids, chaetoglobosins A (**8**), C (**9**), and D (**10**), and aureochaeglobosins B (**11**) and C (**12**), and two azaphilones, chaetoviridin A (**13**), and chaetomugilin A (**14**) (Fig. 1). Herein, the isolation and structural characterization of previously unreported compounds were discussed along with their nitric oxide production inhibitory and anti-angiogenic activities.

## Results

The EtOAc extract of fermented broths of *C. globosum* Km1226 was fractionated and purified sequentially by column chromatography on Sephadex LH-20 and semipreparative HPLC to yield undescribed compounds **1**–**3** as well as eleven known compounds **4**–**14**.

**Fig. 2:**
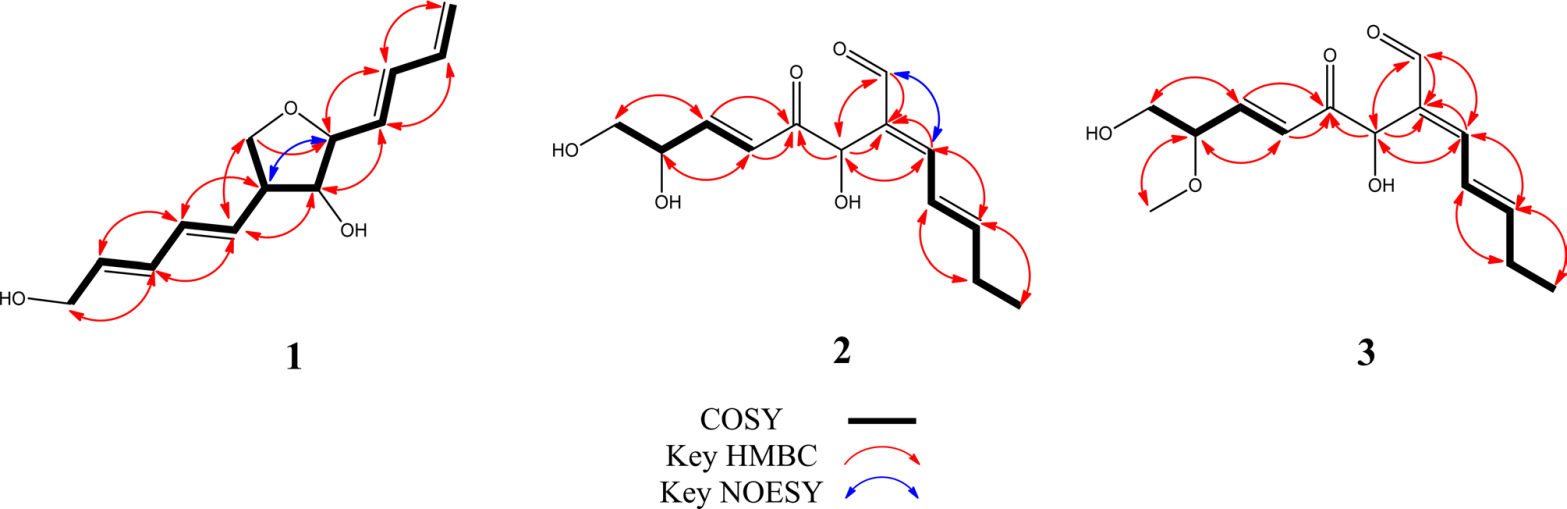
COSY, key HMBC, and NOESY correlations of compounds **1**–**3**

Compound **1** was obtained as white powder, and was deduced to have the molecular formula C_13_H_18_O_3_ as evidenced from a quasi-molecular ion [M + H]^+^ at *m/z* 223.1329 (calcd 223.1334 for C_13_H_19_O_3_) in the HRESIMS, supported by analysis of ^13^C NMR data (Table 1), indicating 5 degrees of unsaturation. Its IR spectrum displayed absorptions at 3357 cm^− 1^, indicating the presence of a hydroxy group. Analysis of ^1^H NMR and HSQC spectra of **1** indicated signals for six mutually *trans*-coupled olefinic protons at *δ*_H_ 6.31 (1H, dd, *J* = 15.0, 10.2 Hz, H-3), 5.72 (1H, dd, *J* = 15.0, 7.2 Hz, H-4), 5.61 (1H, dd, *J* = 14.4, 7.8 Hz, H-8), 6.20 (1H, dd, *J* = 14.4, 10.2 Hz, H-9), 6.23 (1H, ddt, *J* = 15.0, 10.2, 1.2 Hz, H-10), and 5.76 (1H, dt, *J* = 15.0, 5.4 Hz, H-11), two mutually coupled carbinoyl methines at *δ*_H_ 4.06 (1H, m, H-5) and 3.70 (1H, t, *J* = 7.8 Hz, H-6), two oxygenated methylenes at *δ*_H_ 4.08 (2H, dd, *J* = 5.4, 1.2 Hz, H-12) and 4.06 (1H, m, H_a_-13) and 3.70 (1H, t, *J* = 7.8 Hz, H_b_-13), one exomethylene at *δ*_H_ 5.22 (1H, dd, *J* = 16.2, 1.8 Hz, H_a_-1) and 5.09 (1H, dd, *J* = 10.2, 1.8 Hz, H_b_-1), one olefinic proton at *δ*_H_ 6.36 (1H, dt, *J* = 16.2, 10.2 Hz, H-2), and one methine at *δ*_H_ 2.83 (1H, quintet, *J* = 7.8 Hz, H-7) (Table 2). Interpretation of the ^13^C NMR accompanied by HSQC spectra revealed 13 carbon signals attributable to seven olefinic methines at *δ*_C_ 131.8 (C-10), 132.9 (C-8), 133.0 (C-11), 133.3 (C-4), 133.4 (C-9), 134.2 (C-3), and 137.8 (C-2), three methylenes at *δ*_C_ 63.4 (C-12), 72.1 (C-13), and 118.2 (C-1), and three aliphatic methines at *δ*_C_ 52.6 (C-7), 82.6 (C-6), and 86.5 (C-5). Several distinctive signals at *δ*_C_ 63.4 (C-12), 72.1 (C-13), 82.6 (C-6) and 86.5 (C-5) were assigned to oxygen-bearing carbons. Cross-peaks of H-1/H-2, H-2/H-3, H-3/H-4, H-4/H-5, H-5/H-6, H-6/H-7, H-7/H-8, H-8/H-9, H-9/H-10, H-10/H-11, H-11/H-12, and H-7/H-13 in the COSY spectrum together with key cross-peaks of H-3/C-5, H-4/C-6, H-8/C-6, H-9/C-7, H-8/C-13, and H-13/C-5 in the HMBC spectrum (Fig. 2) established the plain structure of **1** as shown. The NOESY correlation of H-5/H-7 (Fig. 2), *J* values (7.8 Hz) of mutually coupled H-7/H-6 and H-6/H-5, and no NOESY correlation of H-7/H-6 and H-6/H-5 established the relative configurations of H-5, H-6, and H-7 to be S^*^, R^*^, and S^*^, respectively. The plain structure of **1** was almost compatible with that of (–)-aureonitol (**4**) except that a terminal CH_3_-12 in **4** was substituted by a hydroxymethyl in **1**. The absolute configuration of **1** of the chiral carbons in the tetrahydrofuran moiety determined as 5*S*, 6*R*, and 7*S* was found to be the same with those of **4** as evidenced from the positive Cotton effect at 220–250 nm in the CD spectra of both **1** and **4** (Fig. S10) and sign of the optical rotational values of **1** ([α]^27^_D_ -9.8) and **4** ([α]^25^_D_ -31.3) in the literature (Nakazawa et al. 2013).

**Table 1:** ^13^ C NMR spectroscopic data for compounds **1**–**3** (δ in ppm)

		1^a^		2 ^a^		3 ^a^
position		δ_C_, mult		δ_C_, mult		δ_C_, mult
1		118.2 d		13.2 q		13.2 q
2		137.8 d		27.7 t		27.7 t
3		134.2 d		153.3 d		153.4 d
4		133.3 d		126.4 d		126.4 d
5		86.5 d		155.9 d		155.9 d
6		82.6 d		137.4 s		137.5 s
7		52.6 d		71.5 d		71.5 d
8		132.9 d		199.1 s		198.9 s
9		133.4 d		125.6 d		127.5 d
10		131.8 d		148.5 d		145.6 d
11		133.0 d		73.1 d		83.1 d
12		63.4 t		66.5 t		64.8 t
13		72.1 t		194.9 d		194.9 d
11-OCH_3_						57.9 q

^a^Measured in methanol-*d*_4_ (150 MHz)

**Table 2:** ^1^ H NMR spectroscopic data for compounds **1**–**3** (δ in ppm, mult., *J* in Hz)

		1 ^a^		2 ^a^		3 ^a^
position		δ_H_, mult (*J* in Hz)		δ_H_, mult (*J* in Hz)		δ_H_, mult (*J* in Hz)
1a		5.22 dd (16.2, 1.8)		1.09 t (7.2)		1.10 t (7.2)
1b		5.09 dd (10.2, 1.8)				
2		6.36 dt (16.2, 10.2)		2.29 qd (7.2, 7.2)		2.30 qd (7.2, 7.2)
				2.29 qd (7.2, 7.2)		2.30 qd (7.2, 7.2)
3		6.31 dd (15.0, 10.2)		6.50 dt (15.0, 7.2)		6.51 dt (15.0, 7.2)
4		5.72 dd (15.0, 7.2)		6.76 ddt (15.0, 12.0, 1.2)		6.78 ddt (15.0, 10.2, 1.2)
5		4.06 m		7.20 d (12.0)		7.22 d (10.2)
6		3.70 t (7.8)				
7		2.83 quintet (7.8)		5.43 s		5.44 s
8		5.61 dd (14.4, 7.8)				
9		6.20 dd (14.4, 10.2)		6.41 dd (15.6, 1.8)		6.38 dd (15.6, 1.2)
10		6.23 ddt (15.0, 10.2, 1.2)		6.98 ddd (15.6, 5.0, 1.8)		6.79 dd (15.6, 6.0)
11		5.76 dt (15.0, 5.4)		4.25 br q (5.0)		3.85 qd (6.0, 1.2)
12a		4.08 dd (5.4, 1.2)		3.51 dd (10.8, 5.0)		3.55 dd (10.2, 6.0)
12b				3.48 dd (10.8, 5.0)		3.49 dd (10.2, 6.0)
13a		4.06 ^b^		9.42 s		9.42 s
13b		3.70 t (7.8)				
11-OCH_3_						3.31 s

^a^Measured in methanol-*d*_4_ (600 MHz)

^b^Signal without multiplicity was overlapped, and was picked up from HSQC experiment

The HRESIMS of compound **2** showed a quasi-molecular ion peak at *m/z* 255.1227 [M + H]^+^ (calcd 255.1232 for C_13_H_19_O_5_) and a deprotonated molecular ion [M − H]^−^ at *m/z* 253.1087 (calcd 253.1081 for C_13_H_17_O_5_), supported by analysis of ^13^C NMR data (Table 1), indicating 5 degrees of unsaturation. Its IR spectrum displayed absorptions at 3365, 1704, and 1672 cm^− 1^, indicating the presence of a hydroxy, an *α*,*β*-unsaturated aldehyde, and an *α*,*β*-unsaturated ketone, respectively. The ^1^H NMR spectrum of **2** showed signals for five mutually-coupled olefinic protons at *δ*_H_ 6.50 (1H, dt, *J* = 15.0, 6.6 Hz, H-3), 6.76 (1H, ddt, *J* = 15.0, 12.0, 1.2 Hz, H-4), 7.20 (1H, d, *J* = 12.0 Hz, H-5), and 6.41 (1H, dd, *J* = 15.6, 1.8 Hz, H-9), and 6.98 (1H, ddd, *J* = 15.6, 5.0, 1.8 Hz, H-10), two carbinoyl methines at *δ*_H_ 5.43 (1H, s, H-7) and 4.25 (1H, br q, *J* = 5.0 Hz, H-11), one aldehydic methine at *δ*_H_ 9.42 (1H, s, H-13), one oxymethylene at *δ*_H_ 3.51 (1H, dd, *J* = 10.8, 5.0 Hz, H_a_-12) and 3.48 (1H, dd, *J* = 10.8, 5.0 Hz, H_b_-12), one methylene at *δ*_H_ 2.29 (2H, qd, *J* = 7.2, 6.6 Hz, H-2), and one methyl at *δ*_H_ 1.09 (3H, t, *J* = 7.2, H-1) (Table 2), which were supported by the COSY assignments of **2**. Interpretation of the ^13^C NMR accompanied by HSQC spectrum revealed 13 carbon signals attributable to seven olefinic methines at *δ*_C_ 125.6 (C-9), 126.4 (C-4), 148.5 (C-10), 153.3 (C-3), 155.9 (C-5), and 194.9 (C-13), two aliphatic methylenes at *δ*_C_ 27.7 (C-2) and 66.5 (C-12), two nonprotonated carbons at *δ*_C_ 137.4 (C-6) and 199.1 (C-8), two aliphatic methines at *δ*_C_ 71.5 (C-7) and 73.1 (C-11), and one methyl at *δ*_C_ 13.2 (C-1). Of all the assigned carbon signals, three distinctive signals at *δ*_C_ 66.5 (C-12), 71.5 (C-7), and 73.1 (C-11) were assigned to be oxygenated carbons. Key cross-peaks of H-5, -7, and -13/C-6, H-5 and -13/C-7, H-5 and -7/C-13, and H-7, -9, and -10/C-8 in the HMBC spectrum (Fig. 1) indicated the gross structure of **2** was as shown. The configurations of ∆^3^, ∆^5^, and ∆^9^ were deduced to be all *E* forms based on a coupling constant of H-3/H-4 (*J* = 15.0 Hz), a key NOESY correlation of H-5/H-13 (Fig. 2), and a coupling constant of H-9/H-10 (*J* = 15.6 Hz), respectively. Due to biogenetic relationship, the relative configuration of C-7 and -11 in **2** was speculated to be the same with those of mollipilin A (**5**). The experimental CD spectrum of **2** was compatible with that of **5** (Fig. S28), and the sign of optical rotational value of **2** ([α]^27^_D_ + 22.2) was the same with that of **5** ([α]^25^_D_ + 39.3) in the literature (Asai et al. 2012). The absolute configurations of C-7 and -11 of **2** were deduced to be *S* and *R*, respectively, as shown in Fig. 1.

The HRESIMS of compound **3** showed protonated molecular ion peaks at *m/z* 269.1383 [M + H]^+^ (calcd 269.1389 for C_14_H_21_O_5_) and a deprotonated molecular ion [M − H]^−^ at *m/z* 267.1242 (calcd 267.1238 for C_14_H_19_O_5_), indicating a molecular formula of C_14_H_20_O_5_ for **3**. When comparing the ^1^H and ^13^C NMR data of **3** with those of **2**, compound **3** almost coincided well with **2** except that the substituent at C-11 in **3** was changed to be a methoxy as judged from the chemical shift of C-11 (*δ*_C_ 73.1) in **2** shifted to *δ*_C_ 83.1 in **3**. A key cross-peak of –OCH_3_/C-11 in the HMBC spectrum corroborated that the additional methoxy group was attached at C-11. The gross structure of **3** was thus determined. Since both the experimental CD spectrum of **3** (Fig. S28) and the sign of optical rotational value of **3** were in compliance with those of **2**, the absolute configuration of **3** was determined to be the same as that of **2**.

(–)-Aureonitol (**4**), a tetrahydrofuran derivative, has been isolated from *C. globosum*, and was found to act like a transcriptional regulator for the biosynthesis of other secondary metabolites in that fungal species (Nakazawa et al. 2013). The structure of mollipilin A (**5**), an epoxide-containing polyketide, was found to exhibit moderate growth inhibitory effects on HCT-116 cells (Asai et al. 2012). Mollipilins E (**6**) and F (**7**), two *spiro*-furan-containing polyketides, accompanied with mollipilin A (**5**) have been isolated from *C. mollipilium* (Asai et al. 2012). Chaetoglobosins A, C, and D (**8**–**10**), three cytochalasan alkaloids, have been isolated from a *Ginkgo biloba*-derived fungal strain *C. globosum* No.04, and were found to exhibit potent anti-fungal effects (Zhang et al. 2019). Aureochaeglobosins B and C (**11** and **12**), two rare aureonitol derivative-fused chaetoglobosins via [4 + 2] cycloaddition, have been isolated from *C. globosum* (Yang et al. 2018). Chaetoviridin A (**13**) and chaetomugilin A (**14**), two chloro-azaphilones, have been found from *C. globosum* by using a molecular epigenetic approach (Wang et al. 2017), and showed significant cytotoxicity against cultured P388 cells and HL-60 cells (Yamada et al. 2008).

**Fig. 3:**
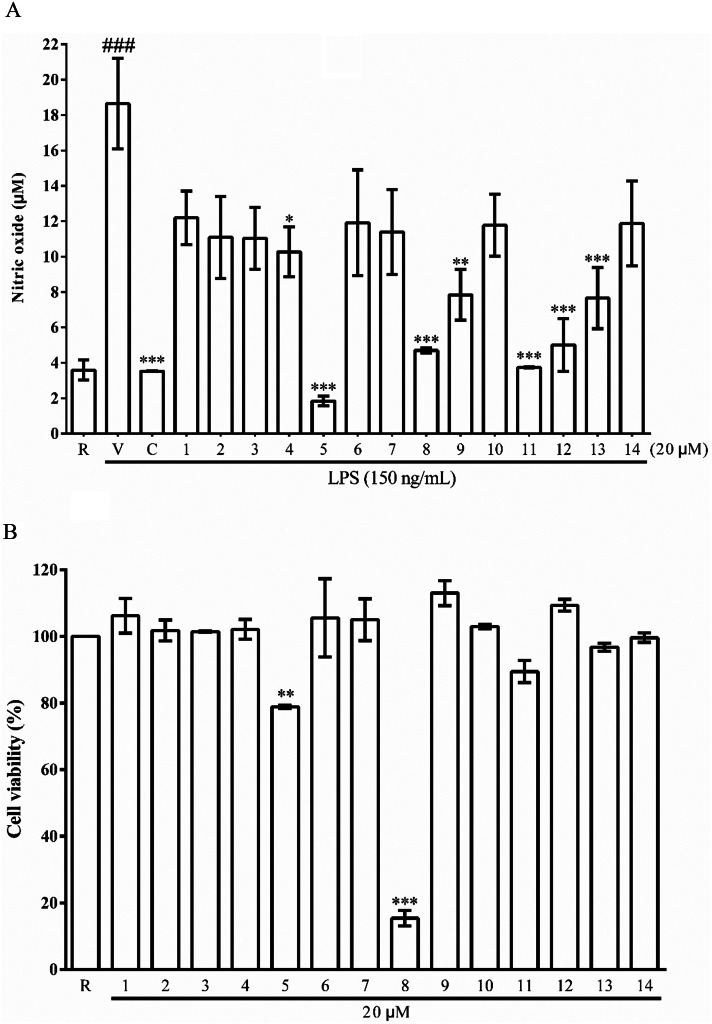
Effects of compounds **1** − **14** on LPS-induced NO production in BV-2 cells (**A**) and their cytotoxicities (**B**). The concentration of test compounds was 20 µM (^*^p < 0.05, ^**^p < 0.01, and ^***^p < 0.001)

Compounds **1**–**14** were tested for anti-inflammatory and anti-angiogenic activities. The anti-inflammatory assay was performed by measuring the amount of nitric oxide (NO) production in lipopolysaccharide (LPS)-induced microgial BV-2 cells. Compounds **1**–**14** inhibited 42.3%, 50.2%, 51.7%, 68.2%, 111.6%, 44.7%, 48.2%, 92.6%, 71.8%, 45.7%, 99.1%, 90.6%, 72.9% and 45.0% of NO production (Fig. 3A), respectively, at a concentration of 20 µM without any cytotoxicity (Fig. 3B) except compounds **5** and **8**. The positive control curcumin exhibited inhibition of NO production 100.4%. Of all the isolates, mollipilin A (**5**), aureochaeglobosins B (**11**) and C (**12**) exhibited significant nitric oxide production inhibitory activity in LPS-induced BV-2 microglial cells with IC_50_ values of 0.7 ± 0.1, 1.2 ± 0.1 and 1.6 ± 0.2 µM, respectively (Table 3). Eendothelial progenitor cells (EPCs) can dictate tumor angiogenesis and cancer progression by activating the angiogenic switch in tumor microenvironment. Therefore, targeting EPCs to develop anti-angiogenic agents is an attractive therapeutic approach for cancer treatment. In this study, it was found that compounds **5**, **8**, **9**, **10**, **11**, and **12** showed promising growth-inhibitory effects on EPCs with IC_50_ values ranging from 1 to 10 µM, with sorafenib as the positive control. As shown in Table 4, chaetoglobosin D (**10**) exhibited the most potent anti-angiogenic activity by suppressing EPCs growth (IC_50_ = 0.8 ± 0.3 µM).

**Table 3:** IC_50_ values of **5**, **11**, and **12** on nitric oxide production inhibitory activities induced by lipopolysaccharide in microglial BV-2 cells

compound	IC_50_ (µM) ^a,b^
**5**	0.7 ± 0.1***
**11**	1.2 ± 0.1**
**12**	1.6 ± 0.2**
Curcumin ^c^	2.7 ± 0.3

**p < 0.01

***p < 0.001

^a^IC_50_ = concentration that reduces NO production by 50%

^b^Asterisks denote significance compared to curcumin (positive control) according to the two-tailed *t*-test

^c^Positive control used in this study

**Table 4:** Anti-angiogenic activity of isolated compounds in human EPCs

compound	IC_50_ (µM) ^a^
**1**	> 50
**2**	> 50
**3**	> 50
**4**	> 50
**5**	5.6 ± 0.1
**6**	> 50
**7**	> 50
**8**	4.6 ± 0.1
**9**	8.1 ± 0.4
**10**	0.8 ± 0.3
**11**	5.2 ± 0.3
**12**	2.9 ± 0.3
**13**	> 50
**14**	> 50
Sorafenib ^b^	4.8 ± 0.3

^a^EPCs were treated with the indicated compounds for 48 h. Anti-angiogenic effects were evaluated in a cell growth assay (*n* = 3). Data are displayed as the mean ± SEM

^b^Positive control used in this study

## Conclusion

In summary, fourteen components **1**–**14**, including three previously undescribed C_13_-polyketides, aureonitol C (**1**), mollipilins G (**2**), and H (**3**), were identified from the cultures of endophytic *C. globosum* Km1226. Mollipilin A (**5**) and chaetoglobosin D (**10**) could be a potential agent for the development of anti-inflammatory and anti-angiogenic leads, respectively, and further studies are still needed to clarify the underlying mechanisms for the biological activities and the structure−activity relationship.

## Methods

### General experimental procedures

The optical rotations were measured on a JASCO P-2000 Digital Polarimeter (JASCO, Tokyo, Japan). The UV spectra were recorded on a Thermo UV-Visible Heλios α Spectrophotometer (Thermo Scientific, Waltham, MA, USA). The IR spectra were obtained on a JASCO FT/IR 4100 spectrometer (Tokyo, Japan). The NMR spectra were recorded at 600 and 150 MHz for ^1^H and ^13^C, respectively, on an Agilent 600 MHz DD2 NMR spectrometer (Agilent, USA). HRESIMS spectra were determined on a Q Exactive Plus Hybrid Quadrupole-Orbitrap Mass Spectrometer (Thermo Fisher Scientific, Bremen, Germany). HPLC separation was performed on a Hitachi HPLC system coupled with a Bischoff RI-8120 RI Detector (Bischoff, Leonberg, Germany) and the Phenomenex Luna column (5 μm PFP column, 100 Å, 250 × 10.0 mm) (Torrance, CA, USA), SunFire column (5 μm C_18_ column, 100 Å, 250 × 10.0 mm) (Milford, Mass, USA) and Phenomenex Gemin column (5 μm C_18_ column, 110 Å, 250 × 4.6 mm) (Torrance, CA, USA). Open column chromatography was performed with Sephadex LH-20 (Amersham Bioscience, Uppsala, Sweden). TLC was carried out with precoated silica gel 60 F254 (Merck, Darmstadt, Germany). Compounds were detected by UV and 10% aqueous H_2_SO_4_ spraying reagent followed by heating at 105 °C for 1 min. The solvents were analytical grade MeOH (Merck, Darmstadt, Germany) and ACN (Merck, Darmstadt, Germany) for HPLC.

### Fungal strain and culture

The fungal strain of *Chaetomium globosum* Km1226 was isolated from fresh leaf of *Atriplex maximowicziana* that were collected from Kinmen County, Republic of China, in May 2018. The fungus was identified according to morphological characteristics and the molecular biology method of amplifying the ITS gene sequence. The sequence data for this strain have been deposited in GenBank with the accession number OQ778818. The purified strain was cultivated in a CMA medium plate (Becton, Dickinson and Company, Sparks, MD, USA) at 29 °C for 14 days. Then agar were cut into small pieces (approximately 0.5 × 0.5 × 0.5 cm^3^) and inoculated into 144 × 250 mL flasks containing 100 mL of PDY medium (containing 10 g dextrose, 2 g peptone and 1 g yeast extract in 1 L distilled water) for liquid state fermentation and 15 × 250 mL flasks containing 10 mL of PDY medium and 20 g brown rice for solid state fermentation which were both sterilized at 121 °C with high pressure. They were fermented for 14 days with shaker equipment at 180 rpm and 28 days at 26 °C, respectively.

### Extraction and isolation of secondary metabolites

The fermentation broth (14.4 L) was filtered, the filtrate was extracted with EtOAc (2 × 14.4 L), and the resulting extract was evaporated under reduced pressure to afford a viscous solid (5.6 g), which was applied to Sephadex LH-20 column chromatography (2.5 cm i.d × 56 cm) and eluted with MeOH obtain eleven main fractions I–X based on TLC analyses. Fraction IV was further purified by semipreparative HPLC using Phenomenex Gemin C_18_ column with 45% MeCN/H_2_O containing 0.1% formic acid (1.0 mL/min) to obtain **1** (4.3 mg, *t*_*R*_ = 10.2 min), and SunFire C_18_ column with 30% MeCN/H_2_O containing 0.1% formic acid (2.0 mL/min) to obtain **2** (7.7 mg, *t*_*R*_ = 20.4 min) and **3** (9.6 mg, *t*_*R*_ = 18.5 min), and Phenomenex Luna PFP column with 80% MeCN/H_2_O (2.0 mL/min) to obtain **4** (0.5 g, *t*_*R*_ = 11.6 min), and SunFire C_18_ column with 30% MeCN/H_2_O (2.0 mL/min) to obtain **5** (59.5 mg, *t*_*R*_ = 30.8 min), Phenomenex Luna PFP column with 65% MeOH/H_2_O (2.0 mL/min) to obtain **6** (11.9 mg, *t*_*R*_ = 19.9 min) and **7** (5.2 mg, *t*_*R*_ = 22.1 min), Phenomenex Luna PFP column with 70% MeOH/H_2_O (2.0 mL/min) to obtain **11** (6.4 mg, *t*_*R*_ = 53.6 min), **12** (12.6 mg, *t*_*R*_ = 39.0 min), **13** (16.3 mg, *t*_*R*_ = 13.8 min) and **14** (7.1 mg, *t*_*R*_ = 61.9 min).

The solid state fermented product (300 g) was lyophilized, ground into powder, and extracted with 1 L of MeOH three times. The combined methanolic extracts were evaporated into a brown residue (18.7 g), which was suspended in 500 mL of H_2_O and partitioned with 500 mL of *n*-hexane three times for deoil, next partitioned with 500 mL of ethyl acetate three times then concentrated under vacuum to dryness (1.2 g). Subsequently, the ethyl acetate extract was redissolved in 10 mL of methanol and applied onto a Sephadex LH-20 column (2.5 cm i.d. × 56 cm) eluted with MeOH obtain eleven main fractions I–IV based on TLC analyses. Fraction III was further purified by semipreparative HPLC using Phenomenex Luna PFP column with 50% MeCN/H_2_O containing 0.1% formic acid (2.0 mL/min) to obtain **8** (103.0 mg, *t*_*R*_ = 24.5 min), **9** (47.3 mg, *t*_*R*_ = 40.6 min) and **10** (5.2 mg, *t*_*R*_ = 13.3 min).

*Aureonitol C* (**1**): white powder; [α]^27^_D_ -9.8 (*c* 0.1, MeOH); UV (MeOH) *λ*_max_ (log *ε*) 232 (4.36), 272 (3.33) nm; IR (ATR) *ν*_max_ 3357, 2926, 2879, 1672, 1646, 1405, 1332, 1210, 1090, 1038 cm^− 1^; HRESIMS *m/z* 223.1329 [M + H]^+^ (calcd for C_13_H_19_O_3_, 223.1334); ^1^H NMR data (methanol-*d*_4_, 600 MHz) see Table 2; ^13^C NMR data (methanol-*d*_4_, 150 MHz) see Table 1.

*Mollipilin G* (**2**): yellowish amorphous; [α]^27^_D_ + 22.2 (*c* 0.1, MeOH); UV (MeOH) *λ*_max_ (log *ε*) 230 (3.74), 280 (3.58) nm; IR (ATR) *ν*_max_ 3365, 2940, 2841, 1704, 1672, 1634, 1452, 1395, 1267, 1198, 1112, 1019 cm^− 1^; HRESIMS *m/z* 255.1227 [M + H]^+^ (calcd for C_13_H_19_O_5_, 255.1232) and 253.1087 [M – H]^−^ (calcd for C_13_H_17_O_5_, 253.1081); ^1^H NMR data (methanol-*d*_4_, 600 MHz) see Table 2; ^13^C NMR data (methanol-*d*_4_, 150 MHz) see Table 1.

*Mollipilin H* (**3**): yellowish amorphous; [α]^27^_D_ + 36.4 (*c* 0.1, MeOH); UV (MeOH) *λ*_max_ (log *ε*) 222 (3.65), 275 (3.39) nm; IR (ATR) *ν*_max_ 3412, 2968, 2944, 2871, 2838, 1708, 1634, 1461, 1367, 1265, 1195, 1056, 1024 cm^− 1^; HRESIMS *m/z* 269.1383 [M + H]^+^ (calcd for C_14_H_21_O_5_, 269.1389) and 267.1242 [M – H]^−^ (calcd for C_14_H_19_O_5_, 267.1238); ^1^H NMR data (methanol-*d*_4_, 600 MHz) see Table 2; ^13^C NMR data (methanol-*d*_4_, 150 MHz) see Table 1.

### Cell culture

The mouse microglial BV-2 cell line was cultured as described previously (Lin et al. 2018). Before experiments, a confluence of 85% of cells were changed to 0.5% FBS media. Thereafter, cells were treated with vehicle or the indicated concentration of compounds **1**–**14** for 15 min and then stimulated with LPS (150 ng/mL) for 24 h. The conditioned medium was freshly collected and frozen at ‒80 ºC.

### Inhibitory activity of nitric oxide (NO) production

Production of NO was evaluated by measuring the levels of nitrite in a conditioned medium as previously described with some modification (Hsieh et al. 2021). The culture supernatants were allowed to react with reconstituted cofactor solution and reconstituted nitrate reductase solution for 1 h at room temperature in the dark according to the instructions of the Nitrate/Nitrite Colorimetric Assay Kit (Cayman). Absorptions were measured at 550 nm using a microplate reader (MRX). Nitrite concentrations were calculated from the standard solutions of sodium nitrite. Curcumin was used as a positive control (Hsiao et al. 2020).

### Cytotoxic activity

The cytotoxicity of compounds addressed in this study against the mouse microglial BV-2 cell line. The cell viability studies were determined by the MTT method. Cells were seeded in 24-well plates at 1 × 10^5^ cells per well and grown for 24 h before use. The seeded cells were first treated with test compounds at 20 µM for 24 h. The final concentration of DMSO in the culture medium of the treated cells was adjusted to less than 0.5% (v/v) to prevent a solvent effect. DMSO was also treated as a vehicle control. Absorbance at 550 nm was obtained by a microplate reader (MRX). All of the experiments were performed in triplicate (Wu et al. 2022).

### Anti-angiogenesis analysis

The methods employed for cell culture and cell growth assessments of human endothelial progenitor cells were conducted as previously reported (Lee et al. 2016; Yang et al. 2019).

### Electronic supplementary material

Below is the link to the electronic supplementary material.

Supplementary Materials 1: Figure S1. ^1^NMR (600 MHz, methanol-*d*_*4*_) spectrum of compound 1.
Figure S2. ^13^C NMR (150 MHz, methanol-*d*_*4*_) spectrum of compound 1.
Figure S3. HSQC spectrum of compound 1.
Figure S4. COSY spectrum of compound 1.
Figure S5. HMBC spectrum of compound 1.
Figure S6. NOESY spectrum of compound 1.
Figure S7. IR (ZnSe) spectrum of compound 1.
Figure S8. HRESIMS spectrum of compound 1.
Figure S9. UV spectrum of compound 1 in MeOH.
Figure S10. ECD spectra of compounds 1and 4.
Figure S11. ^1^H NMR (600 MHz, methanol-*d*_*4*_) spectrum of compound 2.
Figure S12. ^13^C NMR (150 MHz, methanol-*d*_*4*_) spectrum of compound 2.
Figure S13. HSQC spectrum of compound 2.
Figure S14. COSY spectrum of compound 2.
Figure S15. HMBC spectrum of compound 2.
Figure S16. NOESY spectrum of compound 2.
Figure S17. IR (ZnSe) spectrum of compound 2.
Figure S18. HRESIMS spectrum of compound 2.
Figure S19. UV spectrum of compound 2 in MeOH.
Figure S20. ^1^H NMR (600 MHz, methanol-*d*_*4*_) spectrum of compound 3.
Figure S21. ^13^C NMR (150 MHz, methanol-*d*_*4*_) spectrum of compound 3.
Figure S22. HSQC spectrum of compound 3.
Figure S23. COSY spectrum of compound 3.
Figure S24. HMBC spectrum of compound 3.
Figure S25. IR (ZnSe) spectrum of compound 3.
Figure S26. HRESIMS spectrum of compound 3.
Figure S27. UV spectrum of compound 3 in MeOH.
Figure S28. ECD spectra of compounds 2, 3 and 5.

## Data Availability

Data of this study is available with the first author Mr. Chang.
