# Supplementary material for: Chemical constituents from the medicinal herb-derived fungus Chaetomium globosum Km1226
Source: Bot Stud. 2023 Nov 30;64:34. doi: 10.1186/s40529-023-00406-8 (PMC10686906; doi:10.1186/s40529-023-00406-8)

**Supporting Information**

**For**

Chemical Constituents from the Medicinal Herb-derived Fungus *Chaetomium globosum* Km1226

Chia-Hao Chang^1^, George Hsiao^2,3^, Shih-Wei Wang^4,5,6^, Juei-Yu Yen^5,7^, Shu-Jung Huang^1^, Wei-Chiung Chi^7*^, Tzong-Huei Lee1^*^

^1^Institute of Fisheries Science, College of Life Science, National Taiwan University, Taipei 10617, Taiwan.

^2^Department of Pharmacology, School of Medicine, College of Medicine, Taipei Medical University, Taipei 11031, Taiwan.

^3^Graduate Institute of Medical Sciences, College of Medicine, Taipei Medical University, Taipei 11031, Taiwan.

^4^Department of Medicine, MacKay Medical College, New Taipei City 25245, Taiwan. ^5^Institute of Biomedical Sciences, Mackay Medical College, New Taipei City 25245, Taiwan.

^6^School of Pharmacy, College of Pharmacy, Kaohsiung 807378, Taiwan.

^7^Department of Chinese Medicine, MacKay Memorial Hospital, Taipei 10491, Taiwan.

^8^Department of Food Science, National Quemoy University, Kinmen 89250, Taiwan.

**Corresponding Author**

*Phone: +886-82-313-576. Email: joan@nqu.edu.tw.

*Phone: +886-2-3366-1828. Email: thlee1@ntu.edu.tw.

**Table of Contents**

| **Fig. S1** | ^1^H NMR (600 MHz, methanol-*d*_4_) spectrum of compound **1**…. | 3 |
| --- | --- | --- |
| **Fig. S2** | ^13^C NMR (150 MHz, methanol-*d*_4_) spectrum of compound **1**… | 4 |
| **Fig. S3** | HSQC spectrum of compound **1**………………………………. | 5 |
| **Fig. S4** | COSY spectrum of compound **1**……..………………………... | 6 |
| **Fig. S5** | HMBC spectrum of compound **1**……………………………... | 7 |
| **Fig. S6** | NOESY spectrum of compound **1**…..………………….……... | 8 |
| **Fig. S7** | IR (ZnSe) spectrum of compound **1**….………………………... | 9 |
| **Fig. S8** | HRESIMS spectrum of compound **1**…….…………….……… | 9 |
| **Fig. S9** | UV spectrum of compound **1** in MeOH………..……………… | 10 |
| **Fig. S10** | ECD spectra of compounds **1**and **4**……………………………. | 10 |
| **Fig. S11** | ^1^H NMR (600 MHz, methanol-*d*_4_) spectrum of compound **2**…. | 11 |
| **Fig. S12** | ^13^C NMR (150 MHz, methanol-*d*_4_) spectrum of compound **2**… | 12 |
| **Fig. S13** | HSQC spectrum of compound **2**………………………………. | 13 |
| **Fig. S14** | COSY spectrum of compound **2**…………………..…………... | 14 |
| **Fig. S15** | HMBC spectrum of compound **2**……………..………………. | 15 |
| **Fig. S16** | NOESY spectrum of compound **2**………………..…………... | 16 |
| **Fig. S17** | IR (ZnSe) spectrum of compound **2**……………………….…... | 17 |
| **Fig. S18** | HRESIMS spectrum of compound **2**…………………..……… | 17 |
| **Fig. S19** | UV spectrum of compound **2** in MeOH………..……………… | 18 |
| **Fig. S20** | ^1^H NMR (600 MHz, methanol-*d*_4_) spectrum of compound 3…. | 19 |
| **Fig. S21** | ^13^C NMR (150 MHz, methanol-*d*_4_) spectrum of compound 3… | 20 |
| **Fig. S22** | HSQC spectrum of compound 3………………………………. | 21 |
| **Fig. S23** | COSY spectrum of compound 3……..………………………... | 22 |
| **Fig. S24** | HMBC spectrum of compound **3**……………………………... | 23 |
| **Fig. S25** | IR (ZnSe) spectrum of compound **3**….………………………... | 24 |
| **Fig. S26** | HRESIMS spectrum of compound **3**…….…………….……… | 24 |
| **Fig. S27** | UV spectrum of compound **3** in MeOH………..……………… | 25 |
| **Fig. S28** | ECD spectra of compounds **2, 3** and **5**………………………… | 25 |

**Fig. S1** ^1^H NMR (600 MHz, methanol-*d*_4_) spectrum of compound **1**.
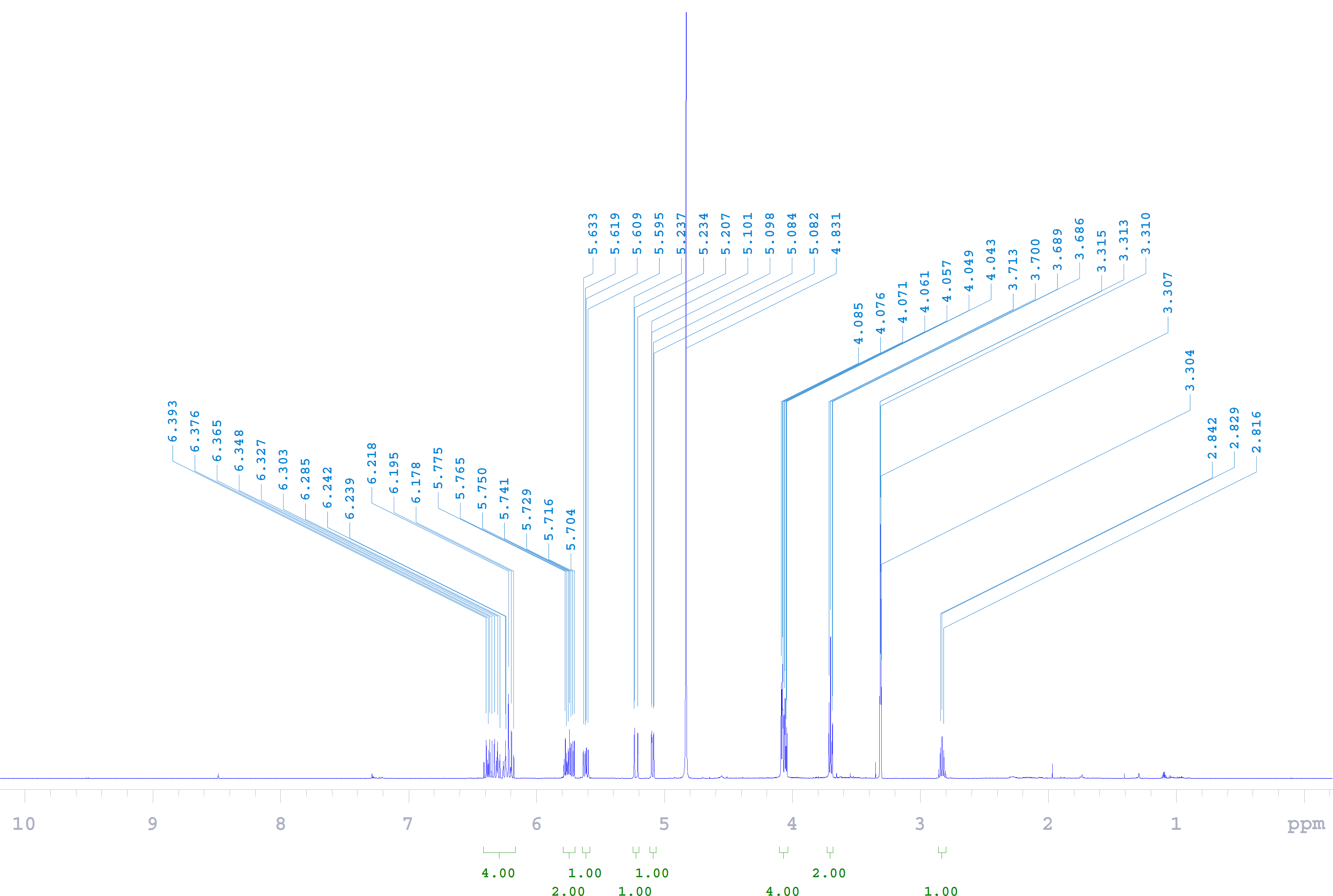

**Fig. S2** ^13^C NMR (150 MHz, methanol-*d*_4_) spectrum of compound **1**.
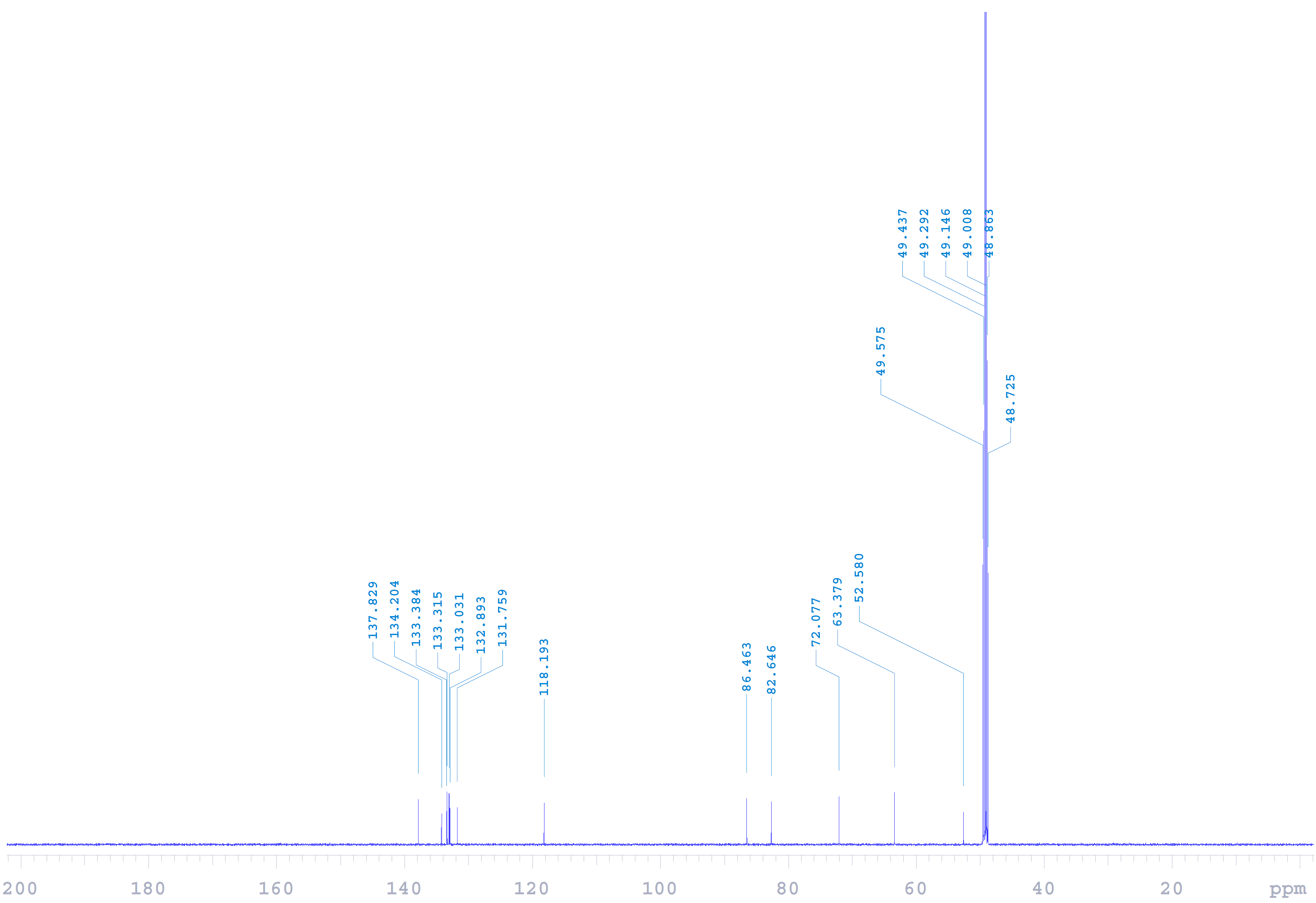


**Fig. S3** HSQC spectrum of compound **1**.
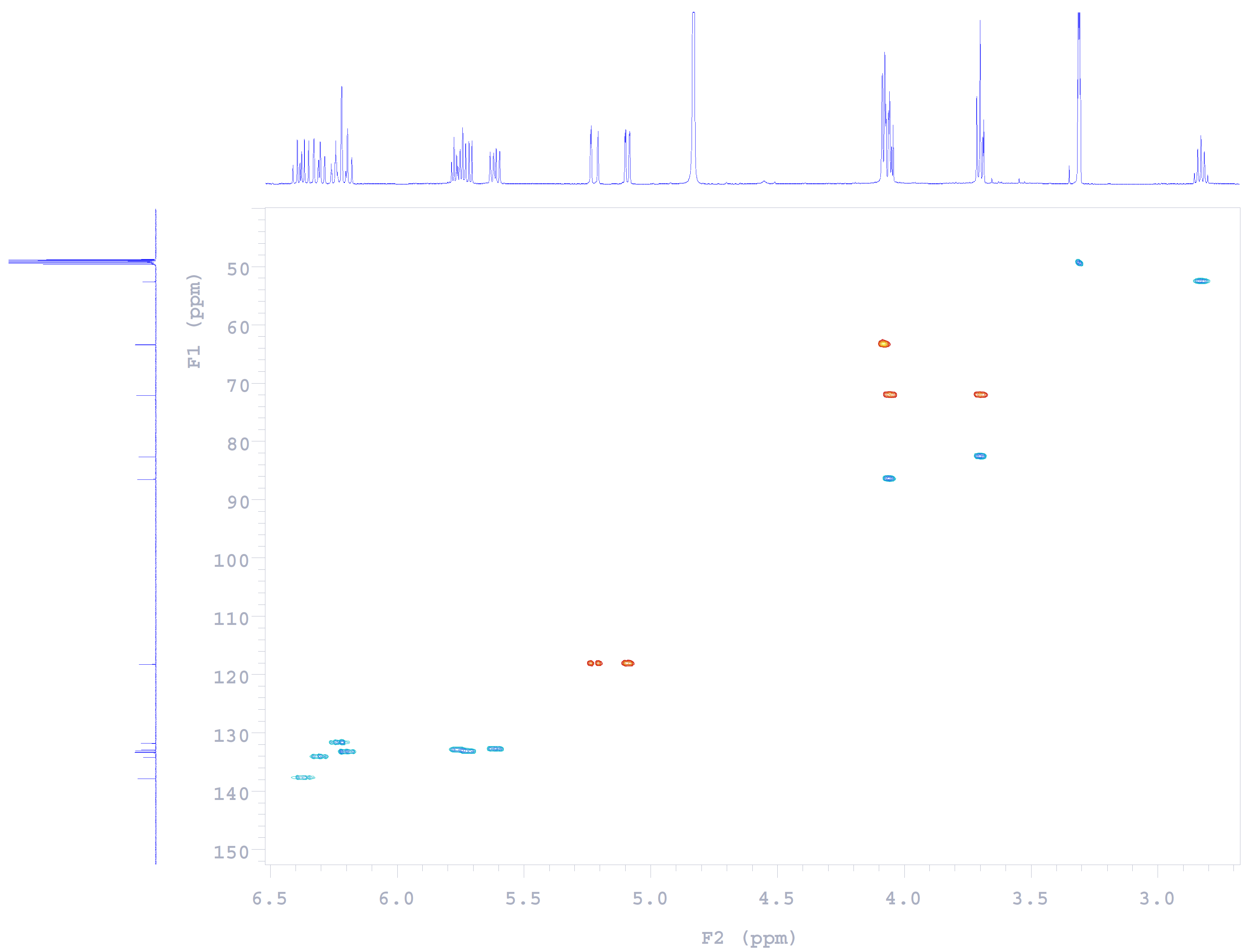


**Fig. S4** COSY spectrum of compound **1**.
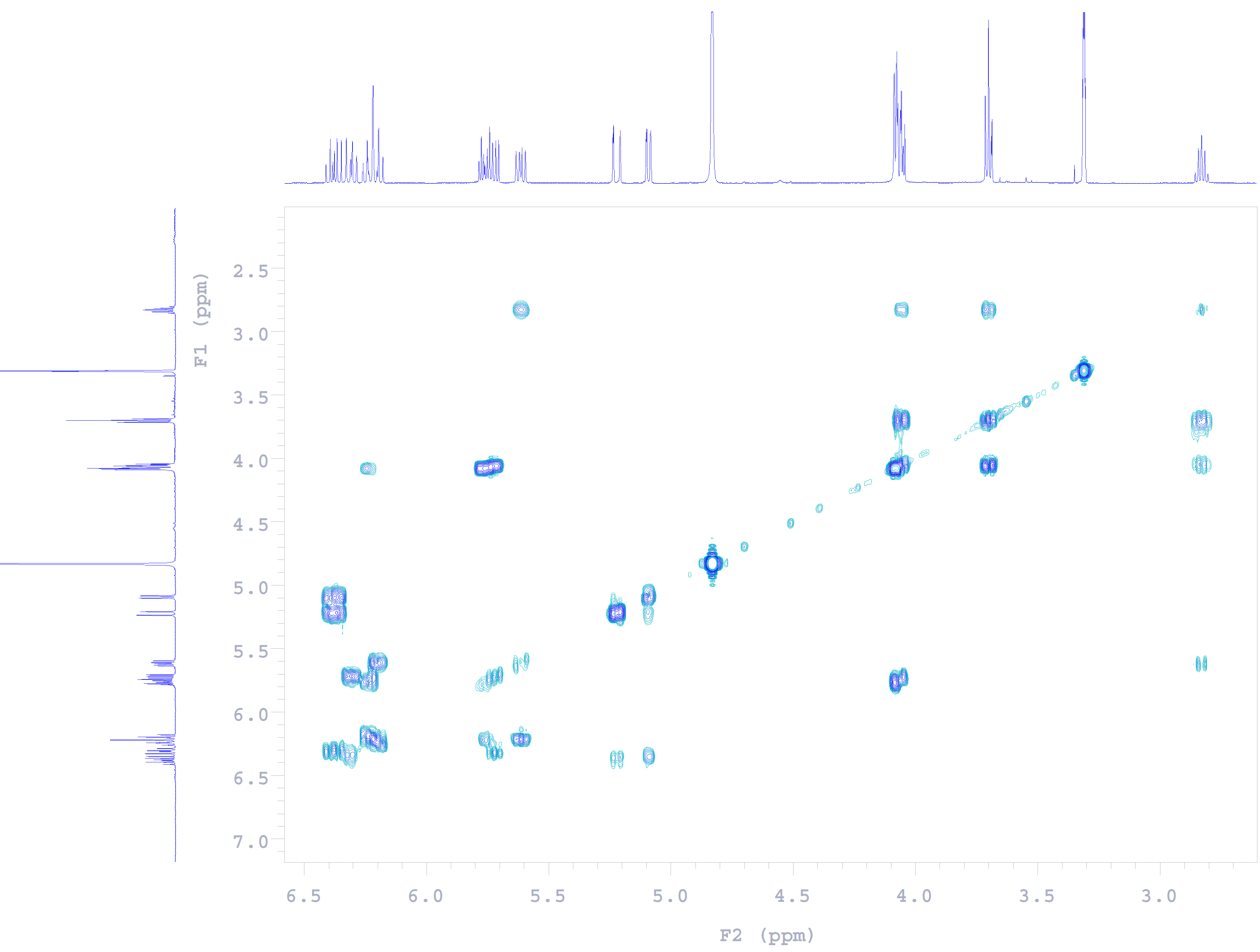

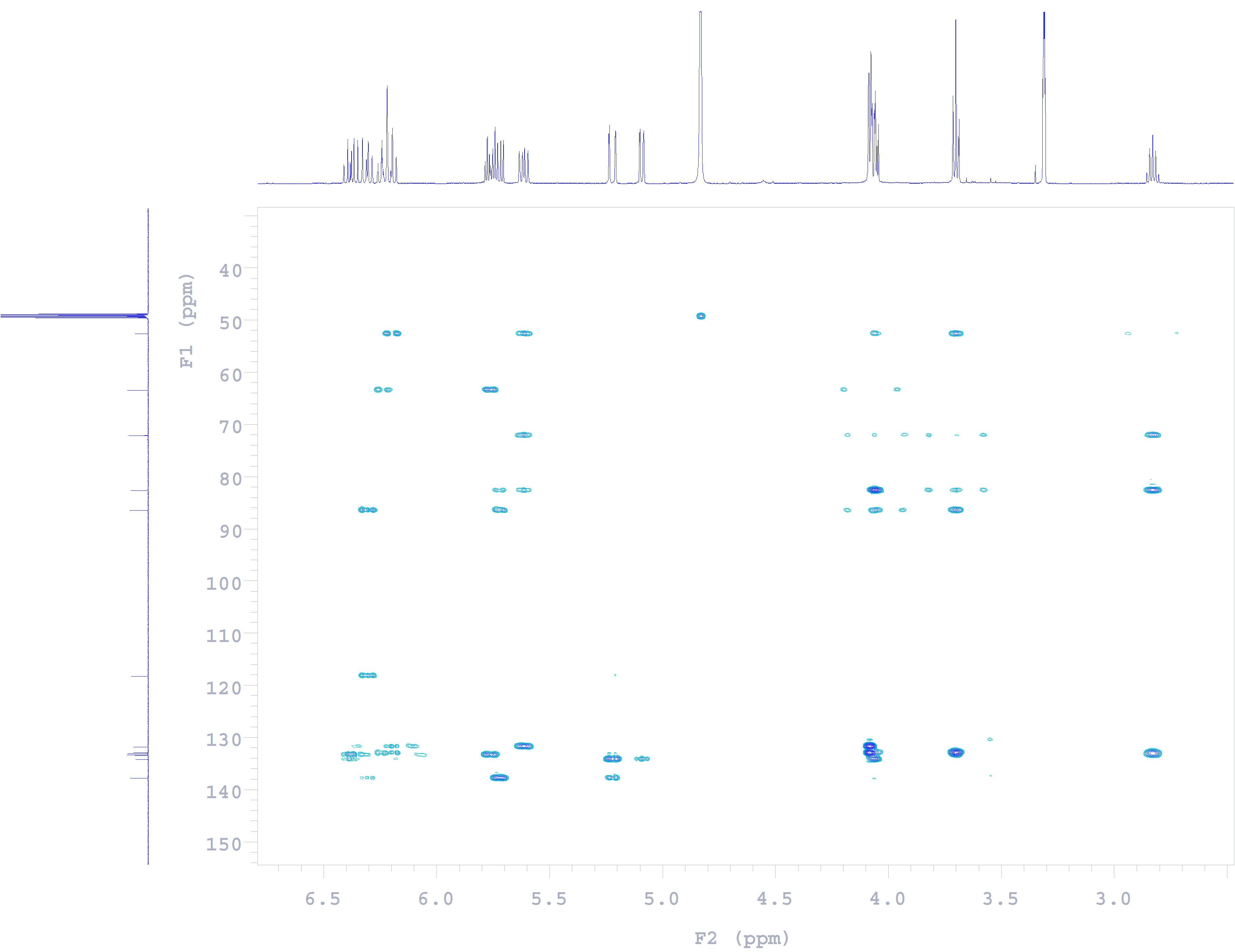
**Fig. S5** HMBC spectrum of compound **1**.

**Fig. S6** NOESY spectrum of compound **1**.
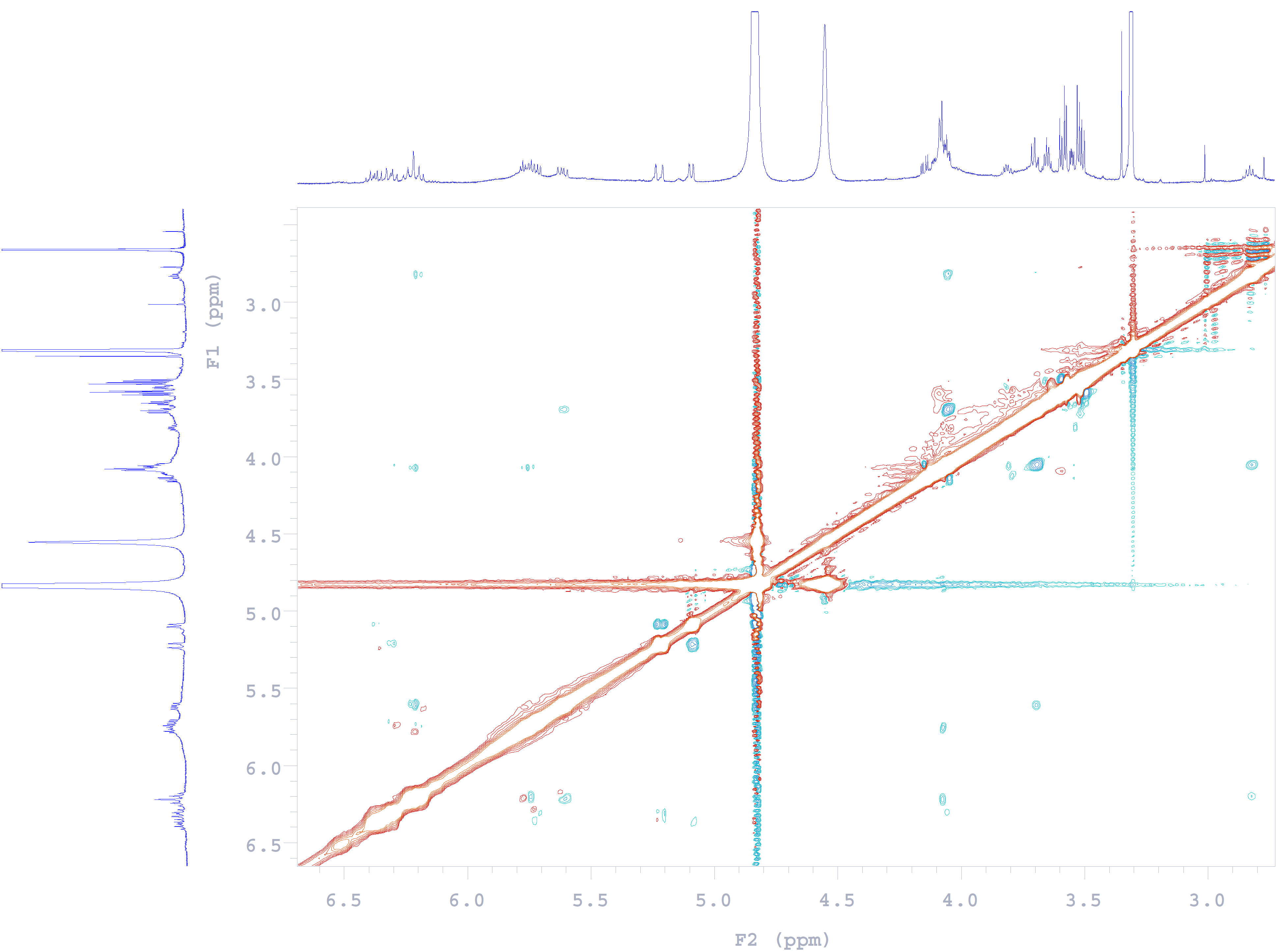

**Fig. S7** IR (ZnSe) spectrum of compound **1**.


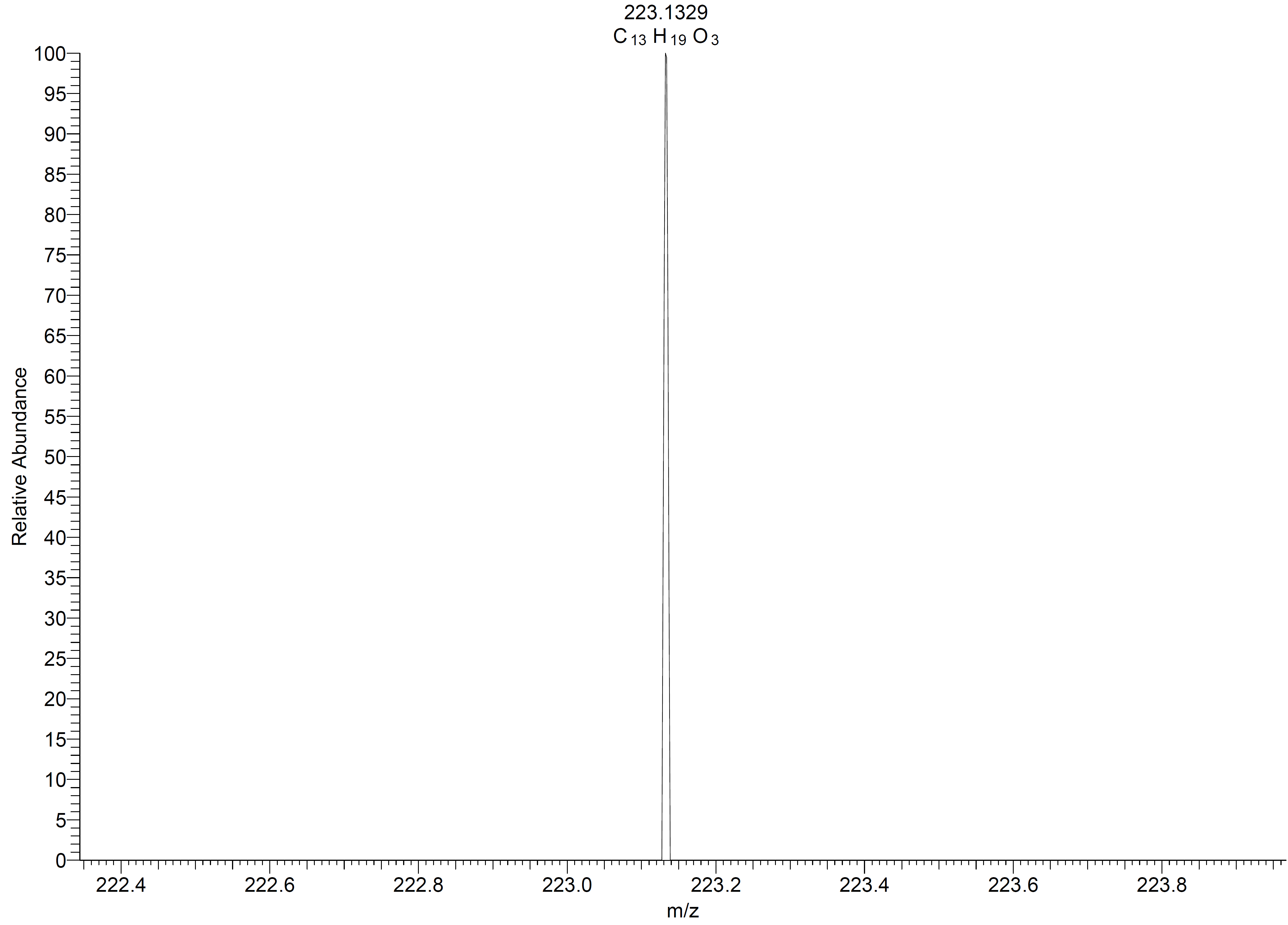


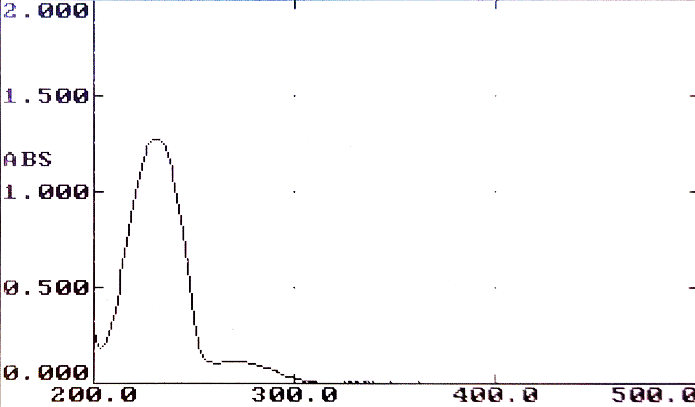


**Fig. S8** HRESIMS spectrum of compound **1**.


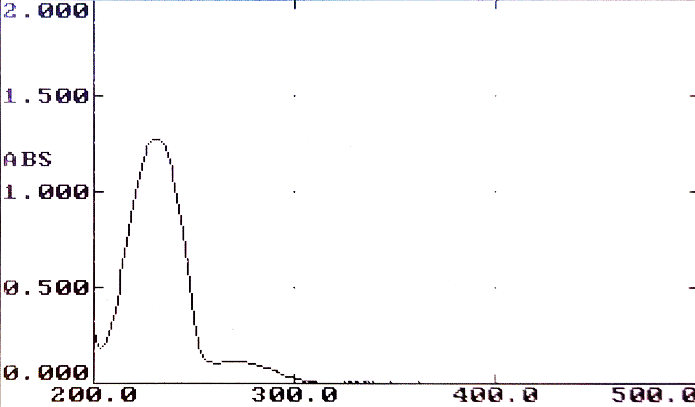

**Fig. S9** UV spectrum of compound **1** in MeOH.





**Fig. S10** ECD spectra of compounds **1**and **4**.

**Fig. S11** ^1^H NMR (600 MHz, methanol-*d*_4_) spectrum of compound **2**.
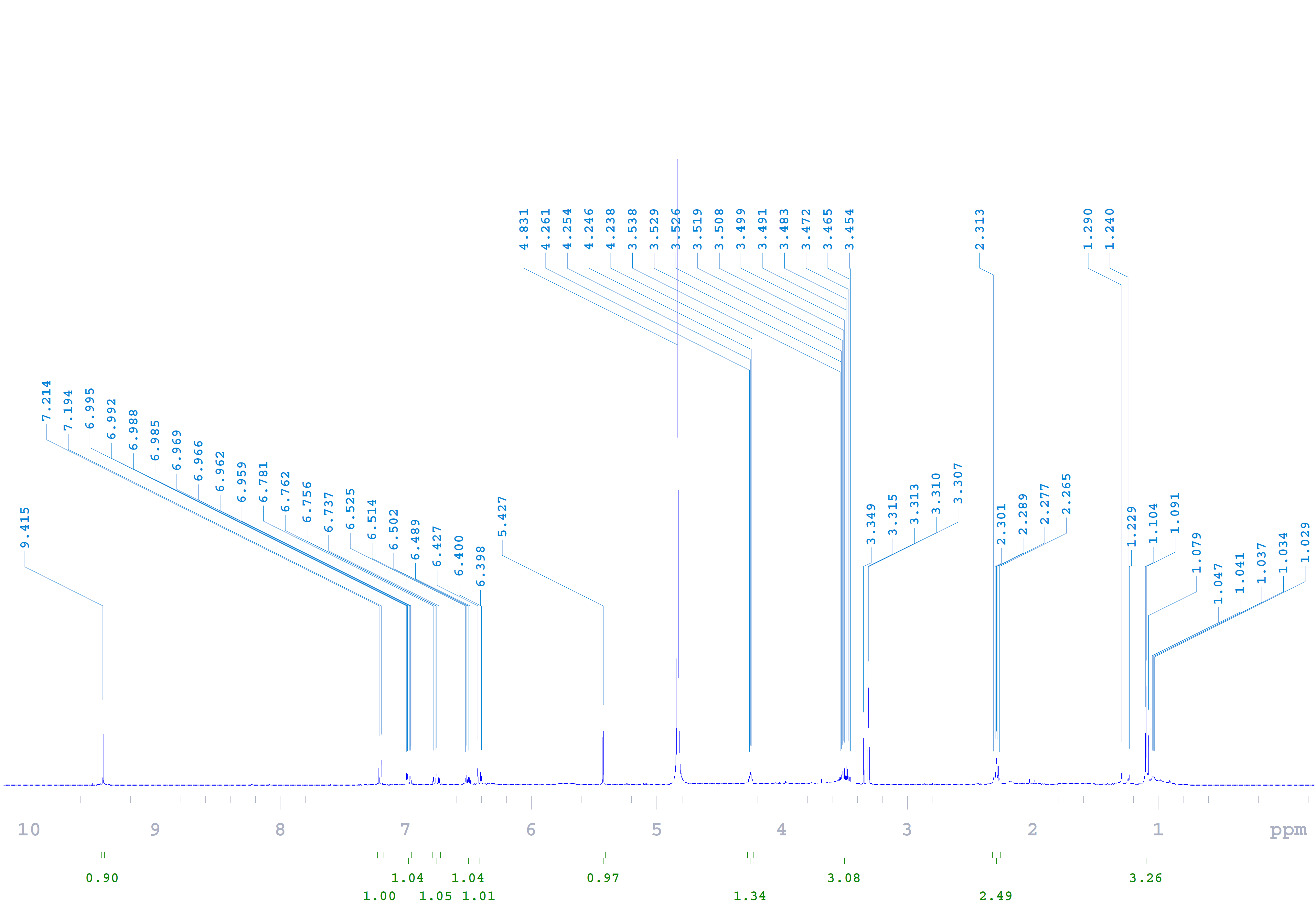


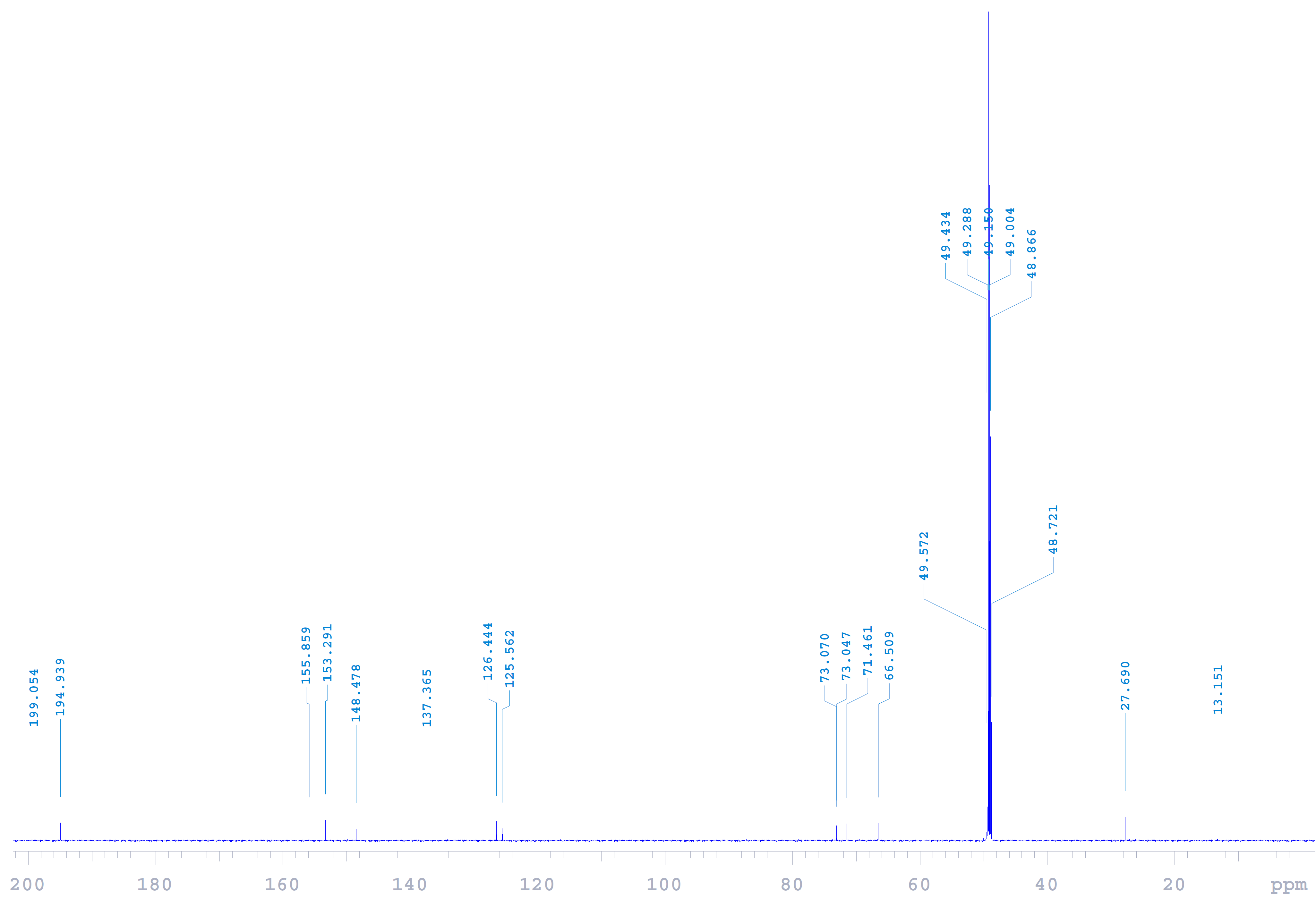
**Fig. S12** ^13^C NMR (150 MHz, methanol-*d*_4_) spectrum of compound **2**.

**Fig. S13** HSQC spectrum of compound **2**
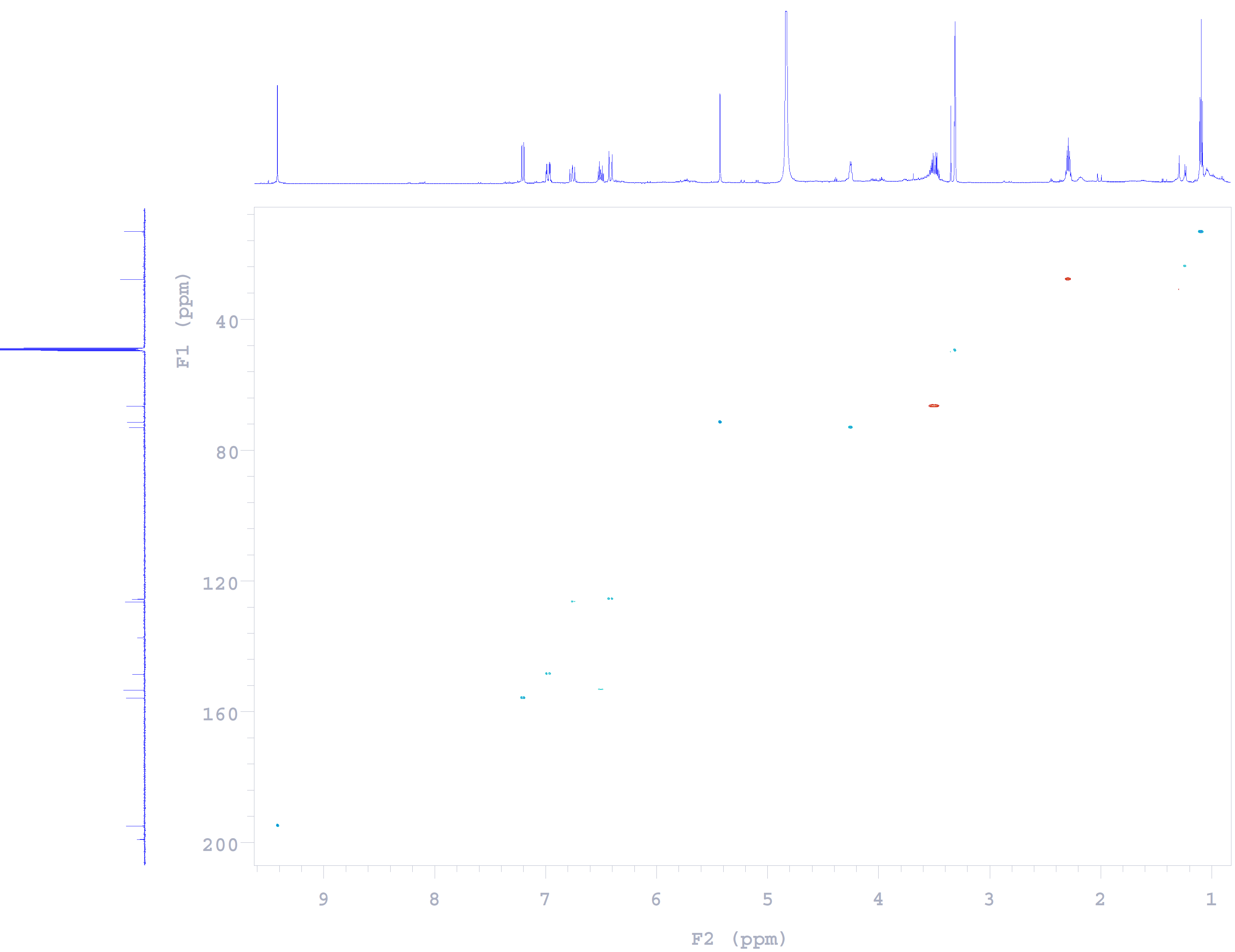
.

**Fig. S14** COSY spectrum of compound **2**.
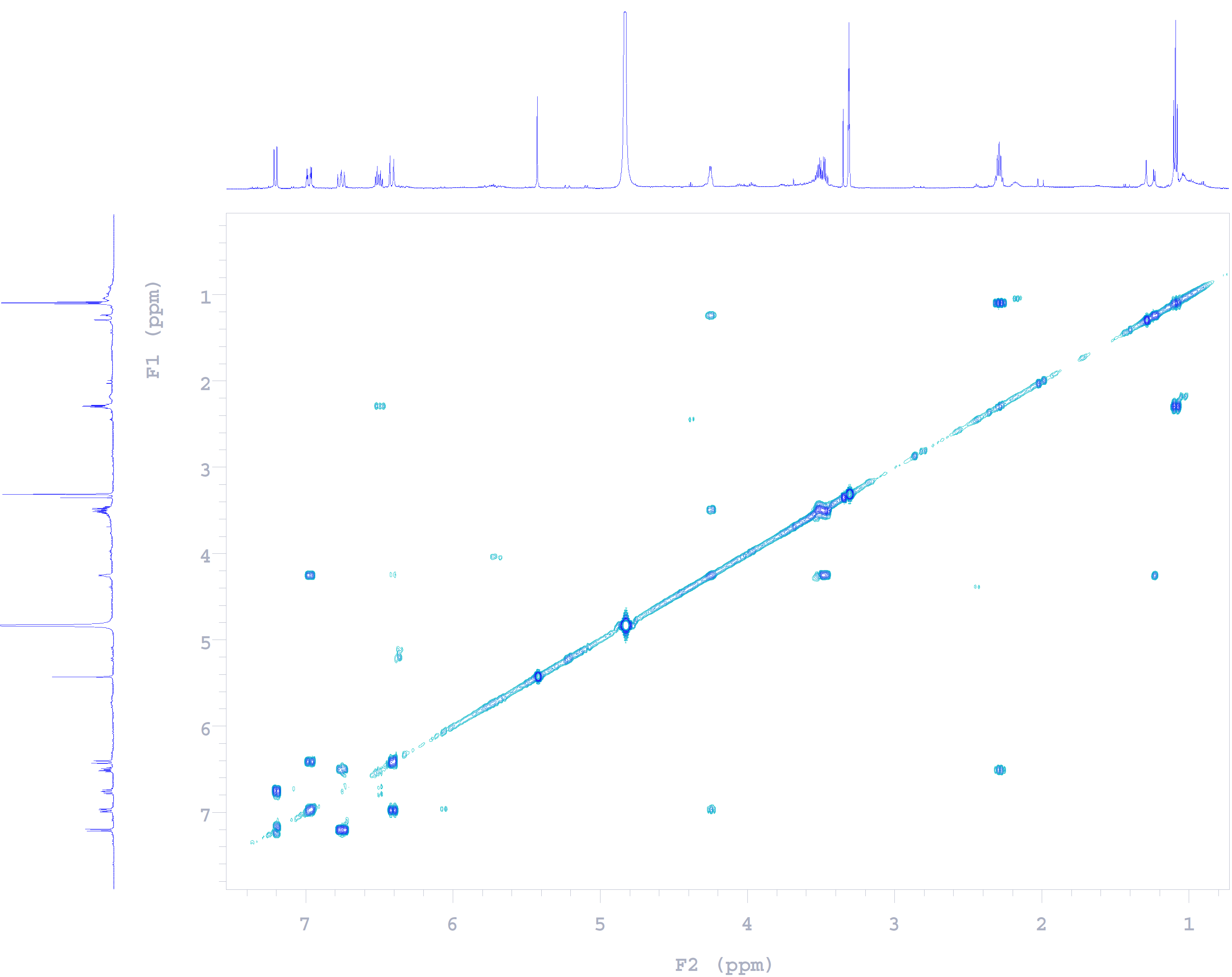


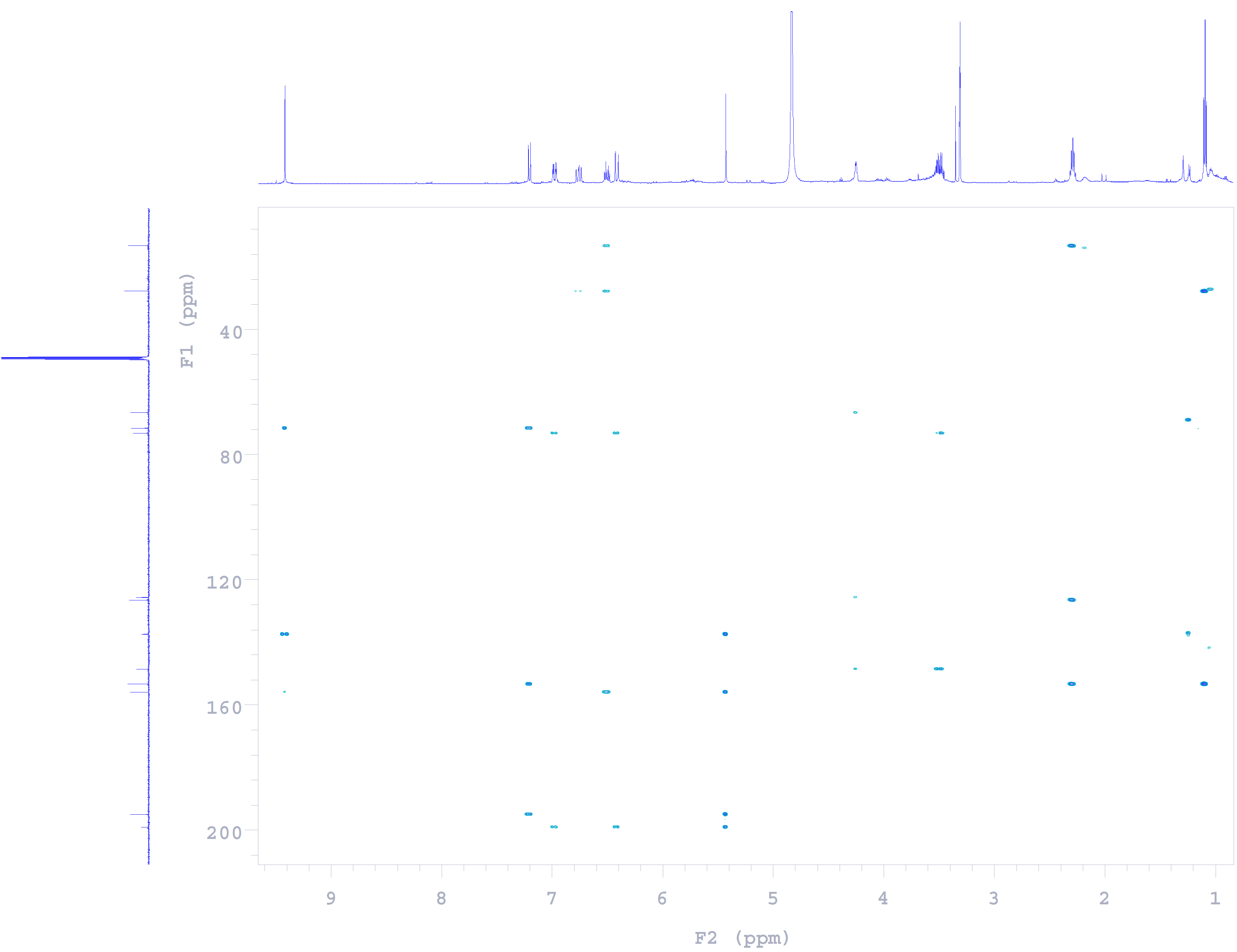
**Fig. S15** HMBC spectrum of compound **2**.

**Fig. S16** NOESY spectrum of compound **2**.
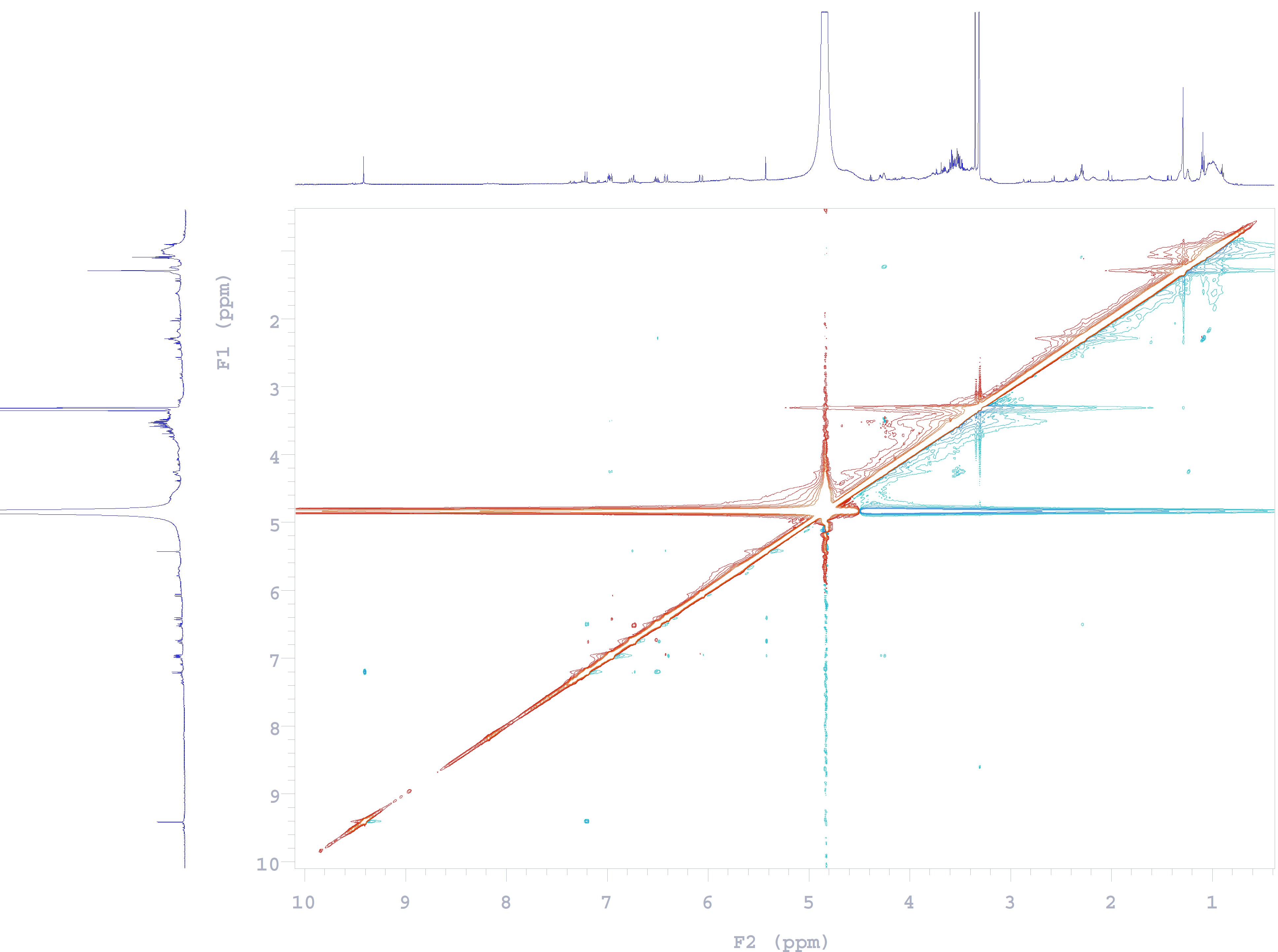

**Fig. S17** IR (ZnSe) spectrum of compound **2**.


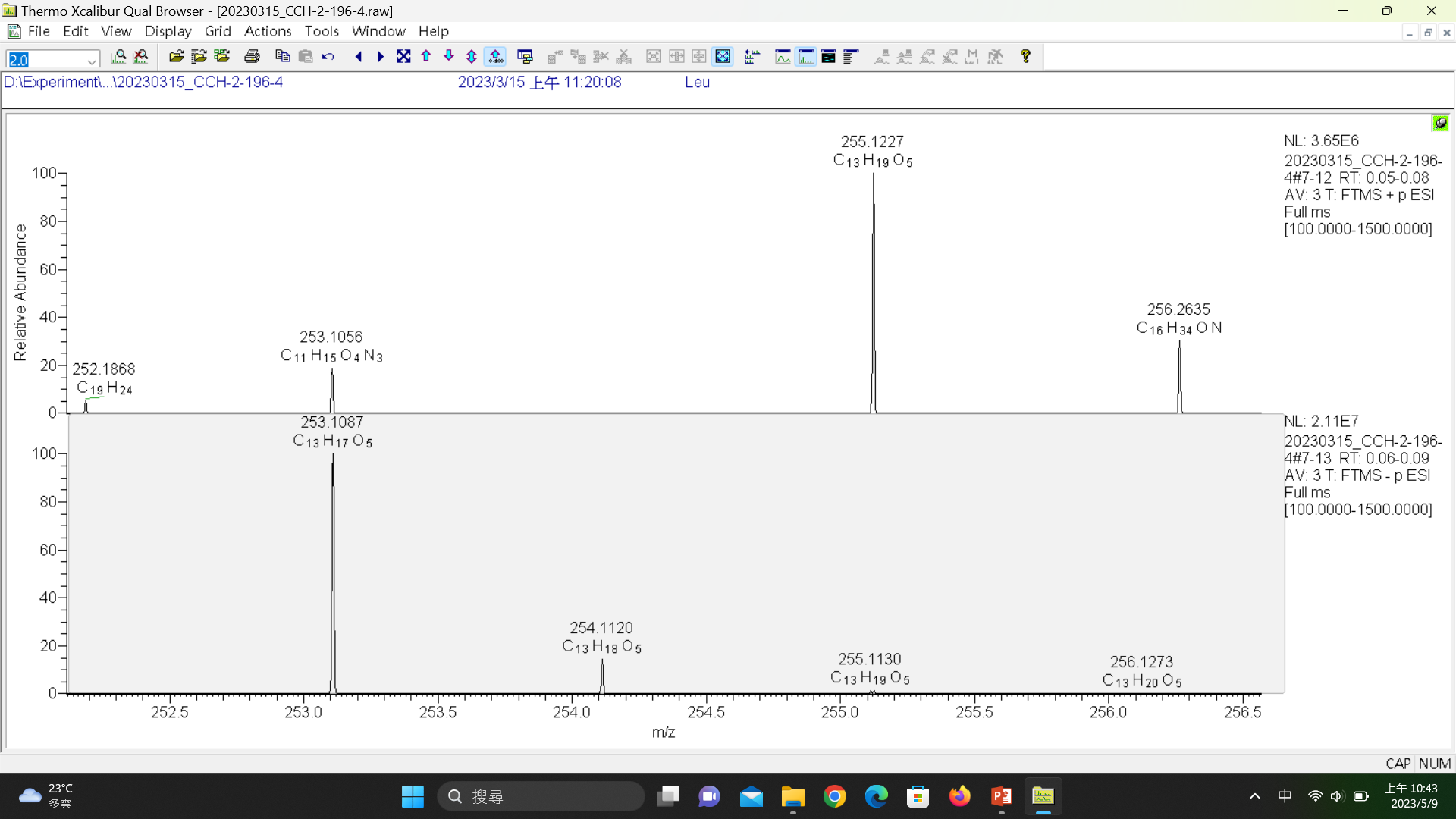


**Fig. S18** HRESIMS spectrum of compound **2**.


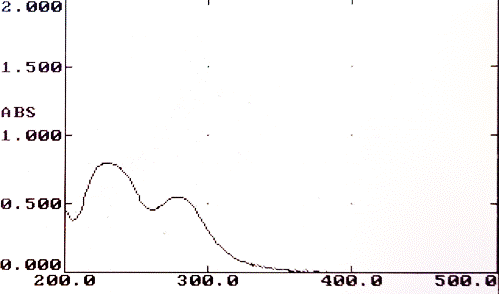


**Fig. S19** UV spectrum of compound **2** in MeOH.

**Fig. S20** ^1^H NMR (600 MHz, methanol-*d*_4_) spectrum of compound **3**.
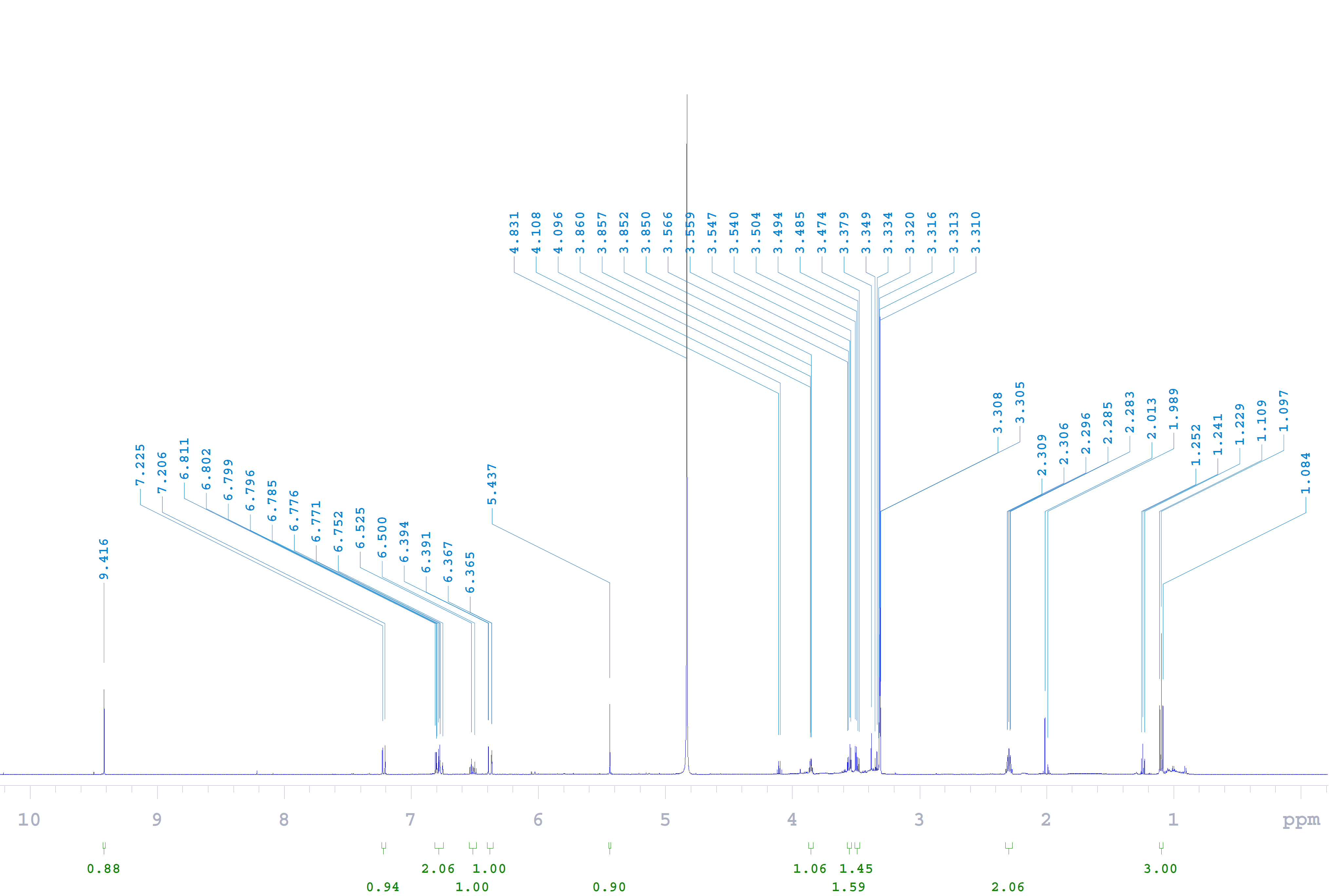


**Fig. S21** ^13^C NMR (150 MHz, methanol-*d*_4_) spectrum of compound **3**.
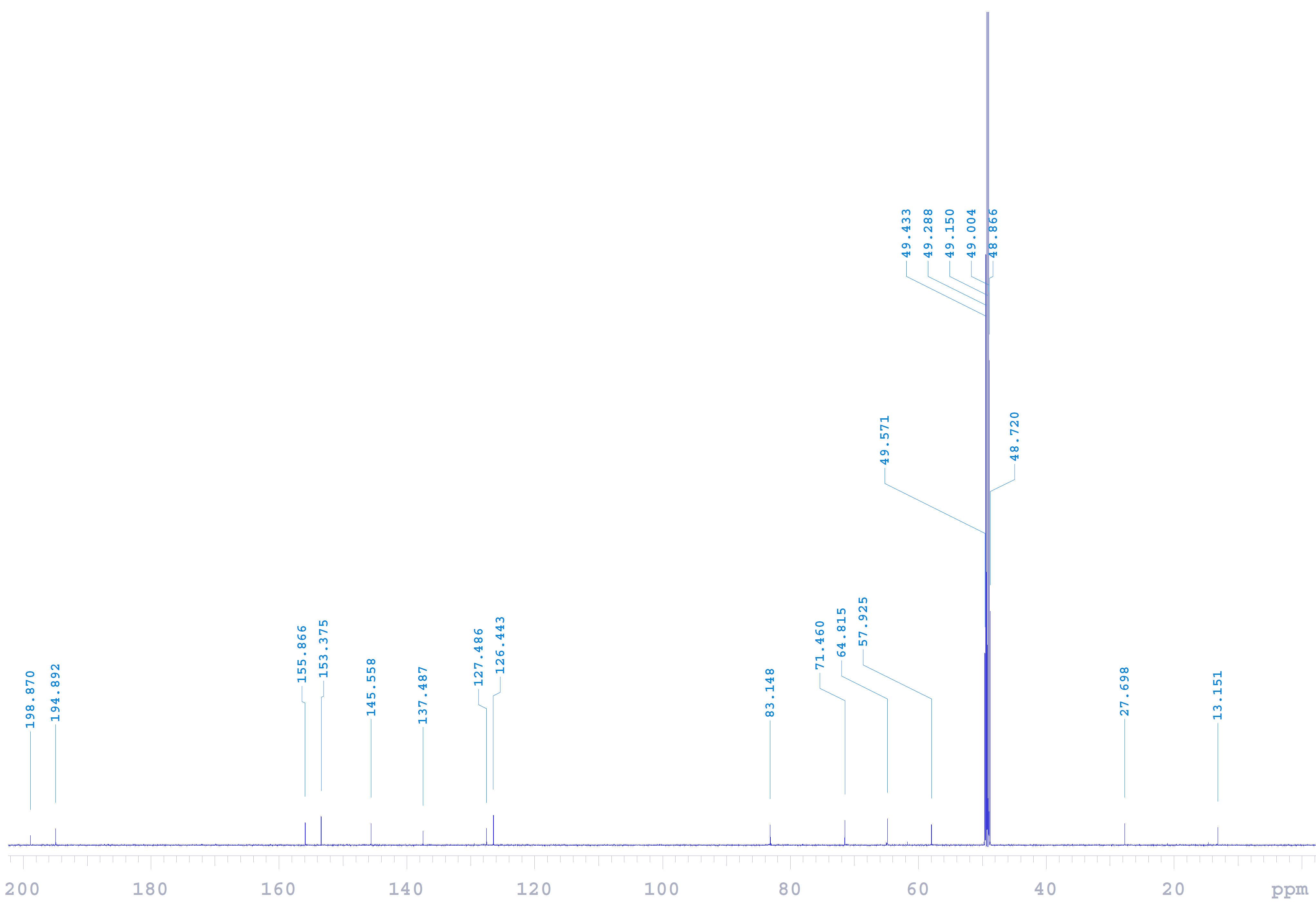


**Fig. S22** HSQC spectrum of compound **3**.
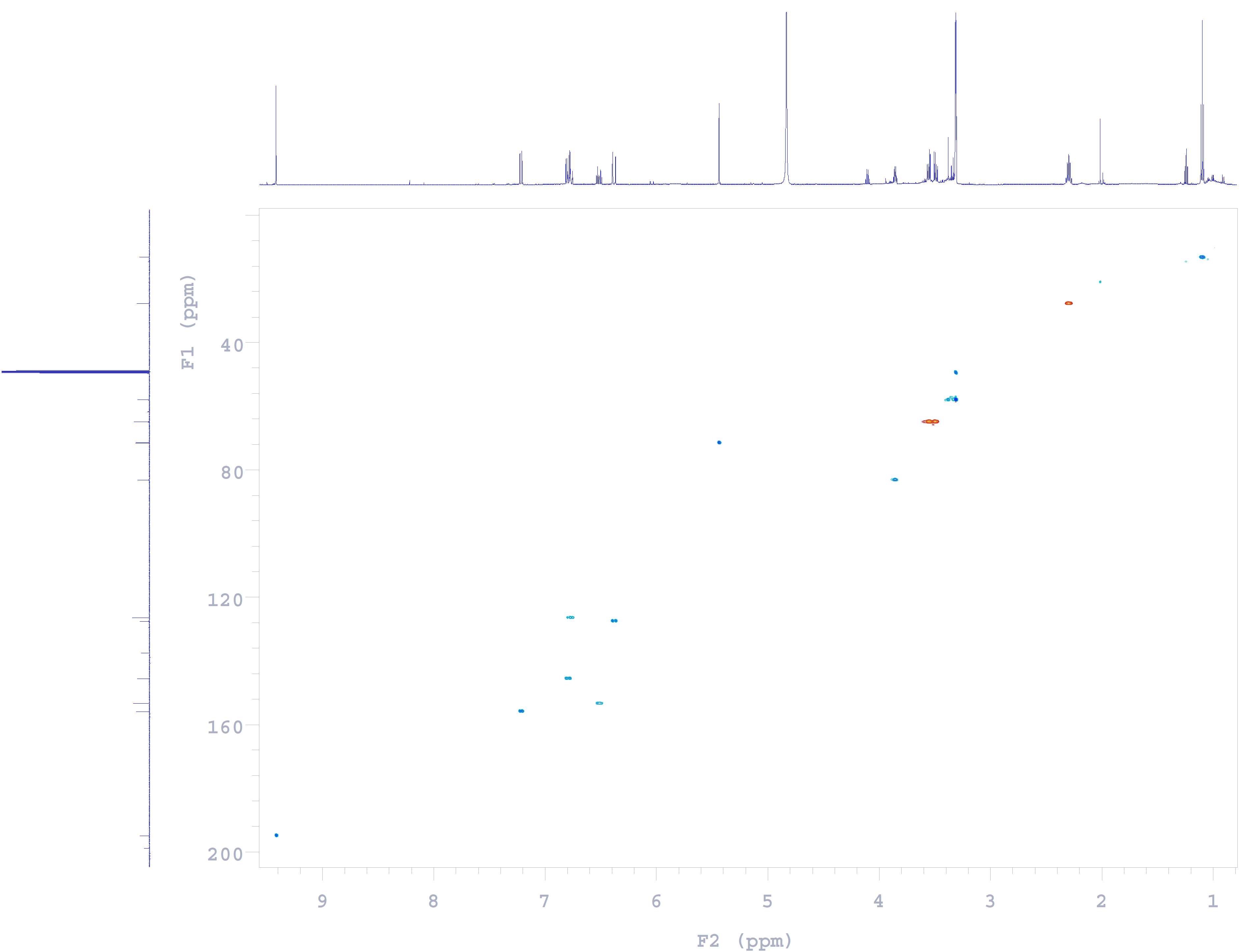


**Fig. S23** COSY spectrum of compound **3**.
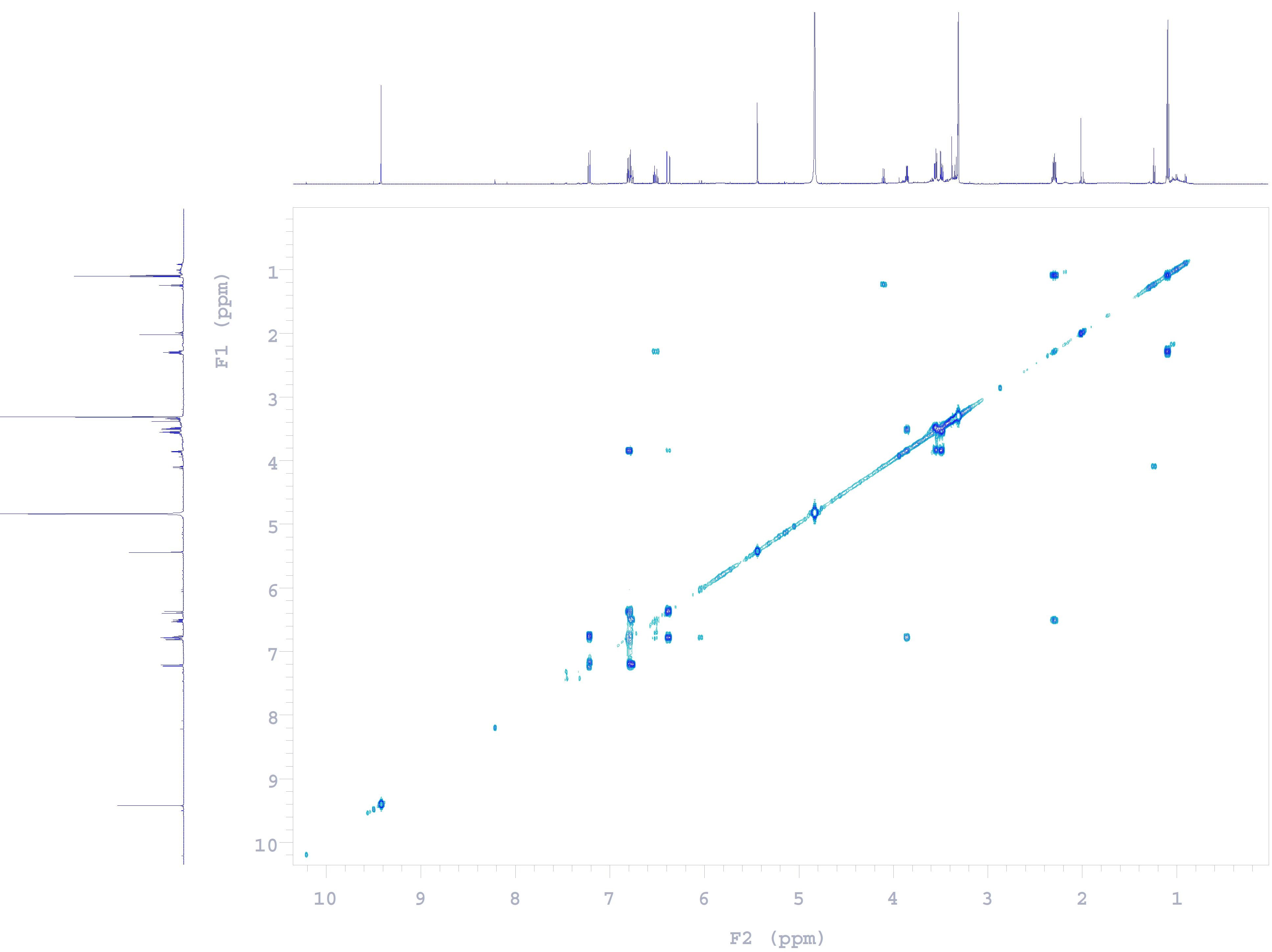


**Fig. S24** HMBC spectrum of compound **3**.
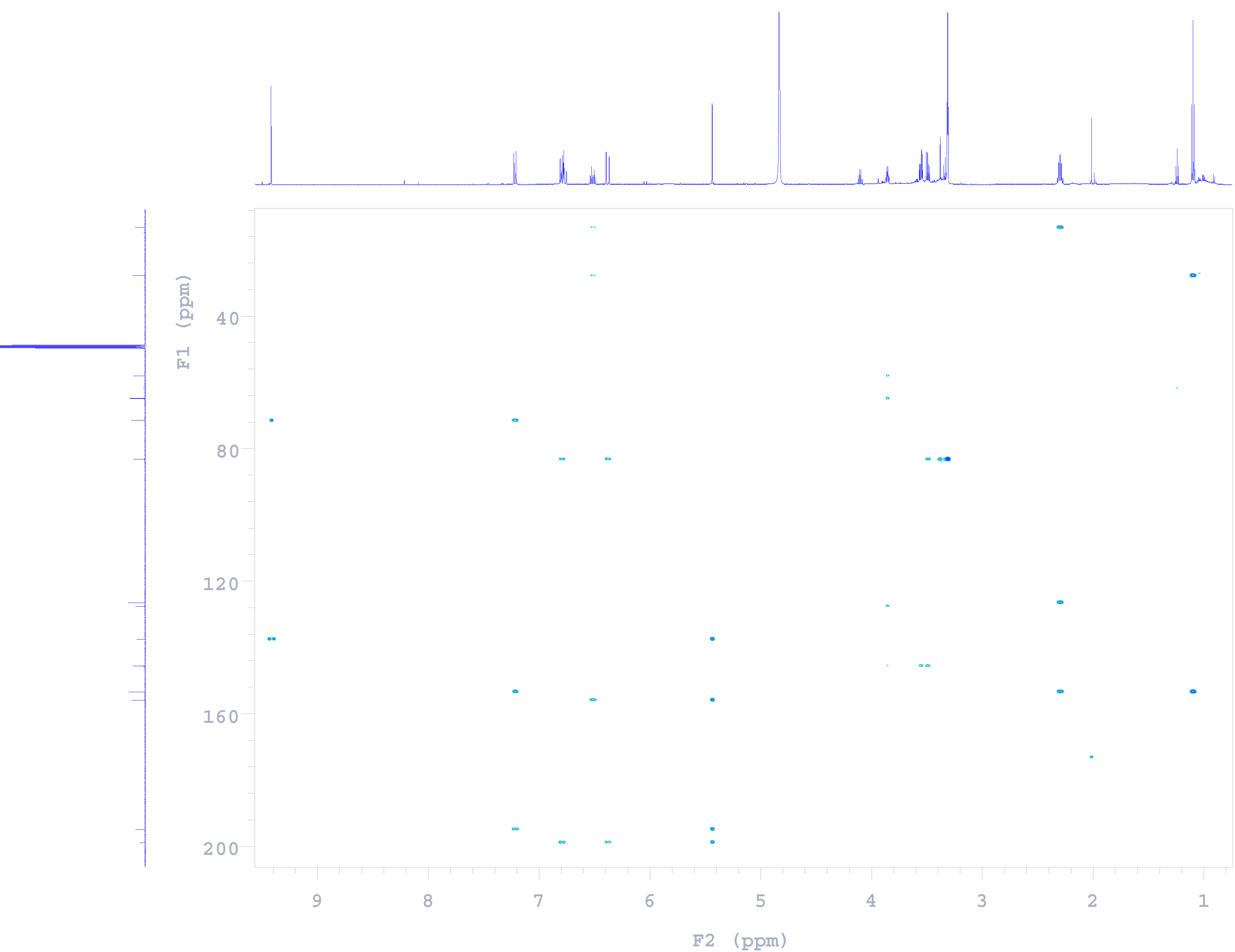

**Fig. S25** IR (ZnSe) spectrum of **3**.


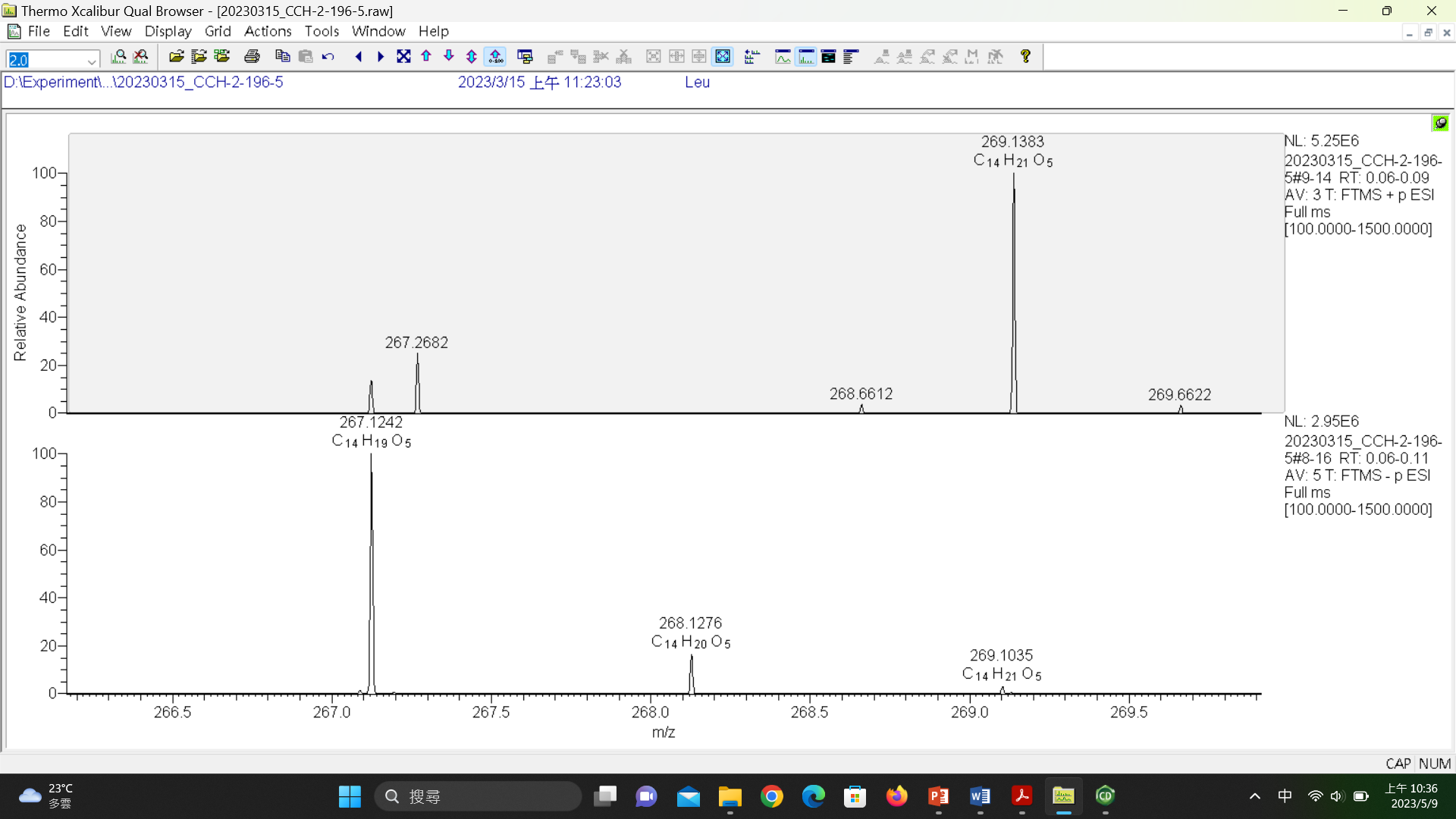


**Fig. S26** HRESIMS spectrum of **3**.

**Fig. S27** UV spectrum of compound **3** in MeOH.





**Fig. S28** ECD spectra of compounds **2, 3** and **5**.


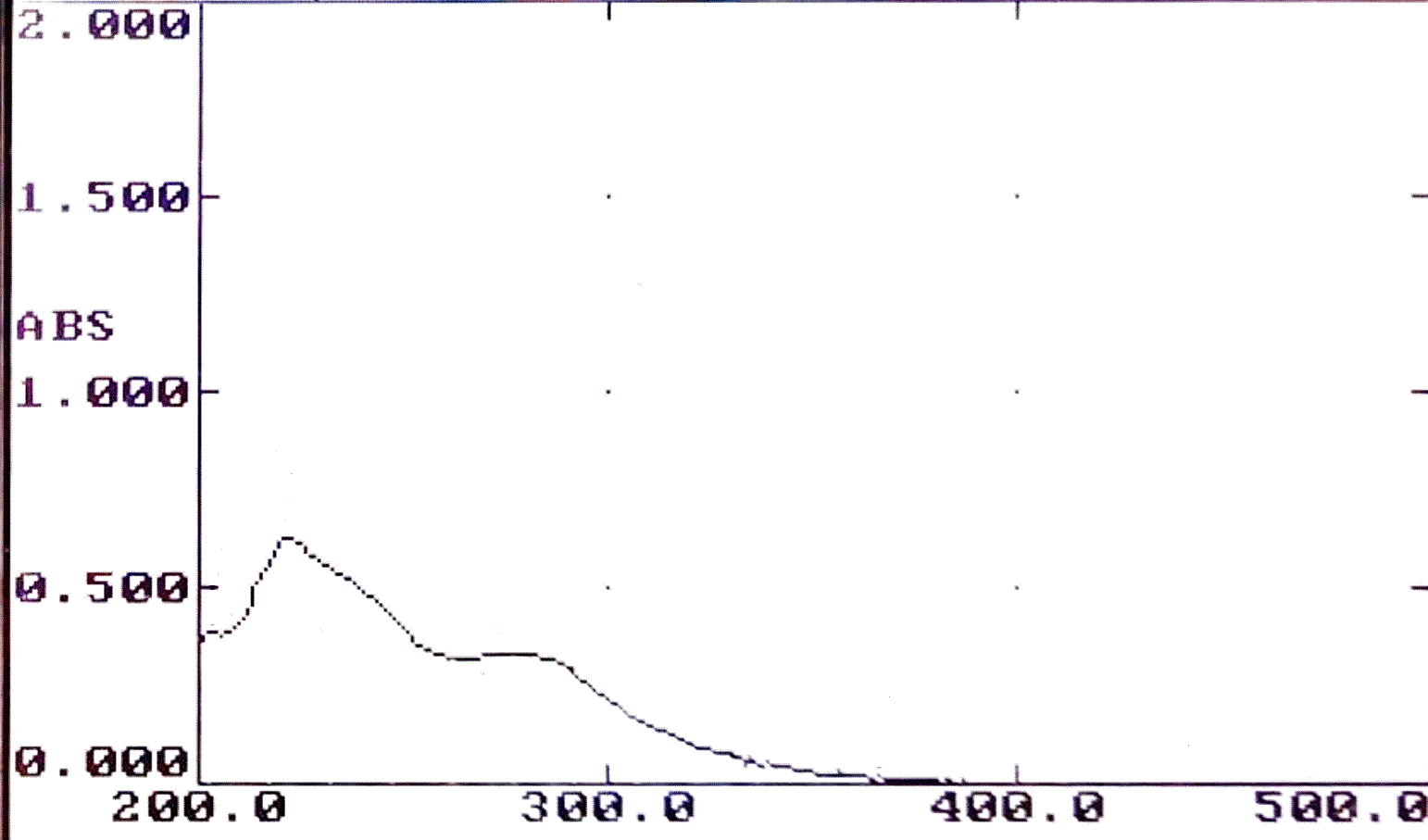

Supplement: Supplementary file 1 — Supplementary Materials 1: Figure S1. 1NMR (600 MHz, methanol-d4) spectrum of compound 1. Figure S2. 13C NMR (150 MHz, methanol-d4) spectrum of compound 1. Figure S3. HSQC spectrum of compound 1. Figure S4. COSY spectrum of compound 1. Figure S5. HMBC spectrum of compound 1. Figure S6. NOESY spectrum of compound 1. Figure S7. IR (ZnSe) spectrum of compound 1. Figure S8. HRESIMS spectrum of compound 1. Figure S9. UV spectrum of compound 1 in MeOH. Figure S10. ECD spectra of compounds 1and 4. Figure S11. 1H NMR (600 MHz, methanol-d4) spectrum of compound 2. Figure S12. 13C NMR (150 MHz, methanol-d4) spectrum of compound 2. Figure S13. HSQC spectrum of compound 2. Figure S14. COSY spectrum of compound 2. Figure S15. HMBC spectrum of compound 2. Figure S16. NOESY spectrum of compound 2. Figure S17. IR (ZnSe) spectrum of compound 2. Figure S18. HRESIMS spectrum of compound 2. Figure S19. UV spectrum of compound 2 in MeOH. Figure S20. 1H NMR (600 MHz, methanol-d4) spectrum of compound 3. Figure S21. 13C NMR (150 MHz, methanol-d4) spectrum of compound 3. Figure S22. HSQC spectrum of compound 3. Figure S23. COSY spectrum of compound 3. Figure S24. HMBC spectrum of compound 3. Figure S25. IR (ZnSe) spectrum of compound 3. Figure S26. HRESIMS spectrum of compound 3. Figure S27. UV spectrum of compound 3 in MeOH. Figure S28. ECD spectra of compounds 2, 3 and 5. [file 40529_2023_406_MOESM1_ESM.docx]
